# Phenotype, donor age and gender affect function of human bone marrow-derived mesenchymal stromal cells

**DOI:** 10.1186/1741-7015-11-146

**Published:** 2013-06-11

**Authors:** Georg Siegel, Torsten Kluba, Ursula Hermanutz-Klein, Karen Bieback, Hinnak Northoff, Richard Schäfer

**Affiliations:** 1 Institute of Clinical and Experimental Transfusion Medicine (IKET), University Hospital Tübingen, Otfried-Müller-Strasse 4/1, Tübingen, D-72076, Germany; 2 Department of Orthopaedic Surgery, University Hospital Tübingen, Hoppe-Seyler-Strasse 3, Tübingen, D-72076, Germany; 3 Institute of Transfusion Medicine and Immunology, German Red Cross Blood Service of Baden-Württemberg-Hessen, Medical Faculty Mannheim, Heidelberg University, Friedrich-Ebert Strasse 107, Mannheim, D-68167, Germany; 4 Department of Neurosurgery, Stanford Institute for Neuro-Innovation and Translational Neurosciences, Stanford University School of Medicine, 1201 Welch Road, Stanford, CA, 94305-5487, USA

**Keywords:** Mesenchymal stromal/stem cells, Age, Gender, Immunomodulation, Phenotype, Differentiation

## Abstract

**Background:**

Mesenchymal stromal cells (MSCs) are attractive for cell-based therapies ranging from regenerative medicine and tissue engineering to immunomodulation. However, clinical efficacy is variable and it is unclear how the phenotypes defining bone marrow (BM)-derived MSCs as well as donor characteristics affect their functional properties.

**Methods:**

BM-MSCs were isolated from 53 (25 female, 28 male; age: 13 to 80 years) donors and analyzed by: (1) phenotype using flow cytometry and cell size measurement; (2) *in vitro* growth kinetics using population doubling time; (3) colony formation capacity and telomerase activity; and (4) function by *in vitro* differentiation capacity, suppression of T cell proliferation, cytokines and trophic factors secretion, and hormone and growth factor receptor expression. Additionally, expression of *Oct4*, *Nanog*, *Prdm14* and *SOX2* mRNA was compared to pluripotent stem cells.

**Results:**

BM-MSCs from younger donors showed increased expression of MCAM, VCAM-1, ALCAM, PDGFRβ, PDL-1, Thy1 and CD71, and led to lower IL-6 production when co-cultured with activated T cells. Female BM-MSCs showed increased expression of IFN-γR1 and IL-6β, and were more potent in T cell proliferation suppression. High-clonogenic BM-MSCs were smaller, divided more rapidly and were more frequent in BM-MSC preparations from younger female donors. CD10, β1integrin, HCAM, CD71, VCAM-1, IFN-γR1, MCAM, ALCAM, LNGFR and HLA ABC were correlated to BM-MSC preparations with high clonogenic potential and expression of IFN-γR1, MCAM and HLA ABC was associated with rapid growth of BM-MSCs. The mesodermal differentiation capacity of BM-MSCs was unaffected by donor age or gender but was affected by phenotype (CD10, IFN-γR1, GD2). BM-MSCs from female and male donors expressed androgen receptor and FGFR3, and secreted VEGF-A, HGF, LIF, Angiopoietin-1, basic fibroblast growth factor (bFGF) and NGFB. HGF secretion correlated negatively to the expression of CD71, CD140b and Galectin 1. The expression of *Oct4, Nanog* and *Prdm14* mRNA in BM-MSCs was much lower compared to pluripotent stem cells and was not related to donor age or gender. *Prdm14* mRNA expression correlated positively to the clonogenic potential of BM-MSCs*.*

**Conclusions:**

By identifying donor-related effects and assigning phenotypes of BM-MSC preparations to functional properties, we provide useful tools for assay development and production for clinical applications of BM-MSC preparations.

## Background

More than 40 years ago Alexander Friedenstein described cells that could be isolated from the bone marrow (BM) and cultured as fibroblastoid colony forming cells *ex vivo*[1,2]. Depicted as mesenchymal stromal cells (MSCs), these cells were shown to support hematopoiesis [3] and osteogenesis [4]. Since then, it has become clear that BM-MSCs offer promising therapeutic potential in regenerative medicine and immunotherapy [5-7]. The most commonly applied technology in research and clinic to isolate and culture BM-MSCs from fresh BM is the removal of non-adherent BM cells and subsequent expansion of the adherent BM-MSCs (BM-MSC preparations) [6,8-10]. Studies reporting on variations in the growth kinetics and gene expression of BM-MSC preparations obtained from different donors [11], variable properties of BM-MSC clones [12], and surface markers identifying BM-MSC subpopulations, such as CD173, CD271, GD2, stage-specific embryonic antigen (SSEA)-4, or mesenchymal stem cell antigen (MSCA)-1 [13-15], highlighted the heterogeneity of MSC preparations. Moreover, their mechanisms of action are still not fully elucidated. Recently, MSC therapies have come under criticism [16] as, despite decades of research, relevant translational questions of MSC biology and function remain unanswered. Many studies reported on the osteogenic, adipogenic and chondrogenic differentiation potential and the immunomodulatory properties of BM-MSCs [15,17,18]; however, controversies prevail about their myogenic or even non-mesodermal differentiation capacity [10,19,20]. *In vivo* efficacy paired with poor survival and homing rate to the damaged tissue points toward mechanisms that most presumably are mediated by factors secreted by BM-MSCs [21,22]. Recently, more straightforward studies reported on gene expression profiling and phenotype of freshly sorted CD271^+^ cells from the BM, and some transcripts appeared to be related to the donor age [23,24]. However, the presence or absence of one single marker as like as CD271 does not sufficiently define all BM-MSC subpopulations within human BM-MSC preparations. Therefore, clarification of how the phenotypes defining BM-MSC subpopulations, as well as age and gender of donors, might affect functional properties of BM-MSCs would mark a significant step forward in our understanding of BM-MSC biology. However, different isolation/expansion technologies/reagents [15] and donor-to-donor variations [11] result in variable fractions of MSC subpopulations per donor/preparation and hamper reliable statistical analyses. Therefore, we obtained BM-MSC preparations from 53 donors of both genders. These multiple BM-MSC preparations were isolated using the most commonly applied technology in research and the clinic [6,8-10], that is, removal of non-adherent BM cells and expansion of the adherent mononuclear BM cells. The BM-MSCs were cultured under identical conditions and analyzed at early passage for phenotype, proliferation capacity, cell size, clonogenic potential, differentiation potential, immunomodulatory potential, secretion of trophic factors, gene expression profile and telomerase activity. Hereby, donor-to-donor variations and variations within the BM-MSC preparations could be identified; however, the great number of BM-MSC preparations allowed statistically robust correlation analyses of phenotype, donor gender and age to functional properties of BM-MSCs.

## Methods

### Isolation and culture of human BM-MSCs

Human BM-MSCs were isolated and cultured as described previously [14]. After written informed consent and approval of the ethical committee of the University Hospital Tübingen, Germany, BM from patients without metabolic or neoplastic diseases was obtained during orthopedic operations. BM mononuclear cells were isolated by density gradient centrifugation on Lymphoflot (Biotest, Dreieich, Germany), washed twice with PBS (Lonza, Walkersville, MD, USA), counted and seeded at a density of 1 × 10^5^ cells per cm^2^ in standard culture medium (SCM), composed of α-MEM (Lonza), 1% penicillin-streptomycin (Lonza) and 10% pooled human blood group AB serum (PHS) (ZKT Tübingen, Germany), to tissue culture flasks (Nunc, Roskilde, Denmark). The average concentrations of sex hormones in the PHS (obtained only from male donors) were in the normal ranges for male individuals above 15 years (testosterone: 16.18 nmol/l; estradiol (eE2): 106.8 pmol/l; estrone (E1): 169.4 pmol/l) and the average bFGF concentration was 75.12 pg/ml. The resulting passage (P)0 cultures were kept under standard culture conditions at 37°C in humidified atmosphere with 5% CO_2_. Non-adherent cells were removed after 24 hours and the adherent cells were cultured in SCM. SCM was changed twice a week until cells reached subconfluency, defined as 90% surface coverage by cells corresponding to 15,000 to 20,000 cells per cm^2^. At this point, the BM-MSCs were detached using Trypsin-EDTA (Lonza), counted using a CASY® cell counter (Roche, Basel, Switzerland) and plated to fresh tissue culture flasks for the next passage (P1) at a density of 1,000 cells per cm^2^.

### Flow cytometry

Flow cytometry analysis of all BM-MSC preparations was performed at the end (subconfluency) of P1 with a FACScan instrument (BD Biosciences, Franklin Lakes, NJ, USA) using BD CellQuest Pro software and the following (secondary) PE-labeled antibodies: anti-CD10, -CD14, -CD19, -CD29, -CD31, -CD34, -CD43, -CD44, -CD45, -CD56, -CD59, -CD71, -CD73, -CD80, -CD86, -CD90, -CD105, -CD106, -CD117, -CD119, -CD130, -CD140a, -CD140b, -CD146, -CD166, -CD273, -CD274, -GD2, SSEA-1, -SSEA-4 and -HLA class I (BD Biosciences); -CD93, -Galectin 1 (R&D Systems, Minneapolis, MN, USA); -CD133 and -CD271 (Miltenyi Biotec, Bergisch Gladbach, Germany); -CD243 (Chemicon (Millipore Corporation), Billerica, MA, USA); -CD173 (AbD Serotec, Puchheim, Germany) and –MSCA-1 (BioLegend, San Diego, CA, USA). PE-conjugated or non-labeled IgG1, -IgG2a, IgG3 and -IgM antibodies (BD Bioscience) were used as isotype matched controls. Secondary antibody was a polyclonal PE-conjugated goat anti-mouse Ig (BD Bioscience). Dead cells were excluded by uptake of 7-Aminoactinomycin D (for gating strategy see Additional file 1: Figure S4, for density plots see Additional file 2: Figure S5). Analysis of percentage of antigen positive cells and fluorescence intensity was performed using FlowJo-7.2.5 software (Tree Star, Ashland, OR, USA). For compensation of unspecific antibody binding, the positivity of the respective matched isotype control was subtracted from all samples.

### Analysis of cell size and PDT

Cell count and analysis of cell size was performed at the end of each passage using a CASY cell counter (Roche). Population doubling time (PDT) during P1 was calculated by the equation PDT = (culture time*ln2)/ln(cell number_harvested_/cell number_seeded_). Seeding density was kept constant at 1,000 cells per cm^2^.

### Colony forming assays

For assessment of colony formation capacity, subconfluent primary BM-MSCs (P0) were trypsinized, counted and seeded at a density of 100, 200 and 500 cells per well (for two MSC populations additional data points with 1,000 cells per well were acquired) in six-well plates at P1 (Nunc, Wiesbaden, Germany). To address possible effects of seeding density on colony formation each MSC preparation was seeded at the aforementioned densities. Cells were cultured during 10 days in MesenCult™ Proliferation Kit (human) medium (Stem Cell Technologies, Vancouver, BC, Canada), then fixed and stained with crystal violet containing 4% formalin. Colonies containing >50 cells were counted microscopically. The number of colonies per 100 seeded cells (percentage colony formation) was calculated for each seeding density for each MSC preparation. These percentages were averaged for each MSC preparation (two experiments for each BM-MSC preparation) and used as one CFU-F data point for the respective MSC preparation for statistical analysis.

### *In vitro* differentiation assays

Functional characterization of BM-MSCs included induction of adipogenic, osteogenic and chondrogenic differentiation *in vitro* as described previously [14]. Briefly, for adipogenic and osteogenic differentiation, BM-MSCs were seeded at a density of 1,000 cells per cm^2^ at P1 and kept under standard culture conditions until reaching subconfluency. Subsequently, either adipogenic differentiation was induced using the hMSC Adipogenic BulletKit (PT-3004, Lonza), or osteogenic differentiation was induced using “osteogenic medium” composed of SCM with 10^-8^ M dexamethasone, 0.2 mM ascorbic acid and 10 mM β-glycerolphosphate (Sigma). After three weeks under differentiation conditions cells were processed for RNA isolation or for lineage specific staining: lipid vacuoles in adipogenic cultures were stained with Oil Red O and calcium deposits of osteogenic cultures with Alizarin Red S, respectively.

For chondrogenic differentiation, 2.5 × 10^5^ BM-MSCs at P1 were kept in micromass pellet cultures for subsequent staining or in monolayer cultures for RNA isolation respectively. Differentiation was induced using the hMSC Chondrogenic Differentiation BulletKit (PT-3003, Lonza), supplemented with TGF-β 3 (PT-4124, Lonza) as a growth factor. After four weeks of differentiation, cells were processed for RNA isolation or frozen sections of fixed pellets were stained with Safranin O.

### Quantitative RT-PCR

For quantitative analysis of lineage specific mRNA indicative for adipogenic, osteogenic and chondrogenic differentiation potential, as well as for expression analysis of *octamer-binding transcription factor (Oct4)*, *Nanog*, *PR domain containing (Prdm)14*, *sex determining region Y-box (SOX)2*, *indoleamine 2,3-dioxygenase (IDO)1 and IDO2* mRNA from differentiated and undifferentiated BM-MSCs was isolated and reversely transcribed to cDNA. To compare *Oct4*, *Nanog*, *Prdm14* and *SOX2* mRNA expression in BM-MSCs to pluripotent stem cells, mRNA was isolated from the human pluripotent teratocarcinoma cell line NCCIT (ATCC-CRL-2073, Ch# 5097030, ATCC, Manassas, VA, USA), and from the human pluripotent embryonic stem cell (hESC) line HUES9 (generously provided by the *Harvard Stem Cell Institute*, Cambridge, MA, USA). Quantitative PCR was performed on resulting cDNA and gene expression was normalized to the expression of the housekeeping gene GAPDH for each sample. Gene induction for differentiation markers was calculated by normalization of the gene expression of differentiated cultures to undifferentiated controls.

#### RNA isolation

RNA from adipogenic, osteogenic and chondrogenic differentiated MSCs, as well as from undifferentiated BM-MSCs (controls) at P1 was isolated using peqGOLD TriFast according to the manufacturer’s instructions.

RNA for analysis of *Oct4*, *Nanog*, *Prdm14*, *SOX2*, *IDO1* and *IDO2* expression was extracted using the RNeasy Mini Kit (Qiagen, Hilden, Germany) according to the manufacturer’s instructions. Remaining genomic DNA was digested using the RNase-Free DNase Set (Qiagen).

RNA concentration was assessed using a NanoDrop photometer (Thermo Scientific, Wilmington, DE, USA. RNA was stored at −80°C for up to three months.

#### Reverse transcription

For analysis by quantitative PCR, 250 μg RNA from each sample was reversely transcribed using the Transcriptor First Strand cDNA Synthesis Kit (Roche) according to the manufacturer’s instructions. Resulting cDNA was stored at −20°C for up to six months.

#### Quantitative PCR

For the adipogenic differentiation markers *LPL* and *PPAR-γ*, the osteogenic markers *AP* and *OPN*, and the chondrogenic markers *SOX9* and *COLL2* as well as for *GAPDH* as housekeeping gene, PCR analysis of cDNA obtained from differentiated and undifferentiated BM-MSC cultures was performed using ready-to-use amplification primer mixes for RT-PCR (search-LC, Heidelberg, Germany) in combination with LightCycler FastStart DNA Master SYBR Green I (Roche) and the LightCycler Instrument (Roche), according to the manufacturer’s instructions. Quantitative PCR assays for *Oct4*, *Nanog*, *Prdm14* and *SOX2* expression of undifferentiated BM-MSCs were performed in the same way.

For the PCR targets *IDO1* and *IDO2*, primers were ordered from Sigma Aldrich (St. Louis, MO, USA) and used in combination with the QuantiTect SYBR Green PCR Kit (Qiagen). Primer sequences were CGGGACACTTTGCTAAAGGCGCT and GGGGGTTGCCTTTCCAGCCAG for *IDO1* and CCTGCAGAGGTCCTGCCAAGGAA and ATGCAGGCTCTCTCCCCCAGG for *IDO2* cDNA. The primer annealing step was performed at 60°C. PCR specificity for *IDO1* and *IDO2* was confirmed by product sequencing (4base lab, Reutlingen, Germany).

PCR results were analyzed by normalizing the expression of each target gene to the expression of the housekeeping gene *GAPDH* in each sample. Gene induction for differentiation markers was calculated by normalization of the gene expression of differentiated cultures to undifferentiated controls.

### T cell proliferation assays

The immunosuppressive properties of BM-MSCs were analyzed in co-culture assays with activated allogeneic T cells. BM-MSCs at P2 were mitotically inactivated by treatment with 40 μg/ml mitomycin C for 30 minutes at 37°C in serum free medium. Thereafter, BM-MSCs were washed three times in SCM and seeded into 96-well plates (Nunc, Roskilde, Denmark) in triplicates at a density of 2 × 10^4^ cells per well. After 24 hours, 1 × 10^5^ PBMNCs, freshly isolated by density-gradient centrifugation from heparinized blood of healthy donors and normalized to the lymphocyte number, were added to the appropriate wells of BM-MSC cultures. T cells were stimulated by addition of 10 μg/ml Phytohaemagglutinin-L (Sigma) and cultured for 72 hours under standard culture conditions. During the last six hours, 100 μM Bromodeoxyuridine (BrdU) labeling solution from the Cell Proliferation ELISA, BrdU (colorimetric) Kit (Roche) was added to the (co-) cultures and cell proliferation was assessed by the subsequent ELISA, using the anti-BrdU antibody provided in the kit according to the manufacturer’s instructions. Photometric light absorption was measured in a plate reader and absorbance values were averaged and normalized to the PBMNC only control of the respective blood donor. Supernatants of T cell proliferation assays were collected and stored at −80°C for subsequent analysis of cytokine production.

### Multiplex analysis of cytokine production

Cytokine production of BM-MSCs alone and of BM-MSC-PBMNC co-cultures was analyzed in supernatants of T cell proliferation assays. Samples of 25 μl cell culture supernatant were analyzed in triplicates using human cytokine/chemokine multiplex kits (Millipore) according to the manufacturer’s instructions. The Kits were composed of beads for detection of IL-2, IL-4, IL-6, IL-8, IL-10, IFN-γ and TNF-α. Analysis was performed using a Luminex® 200 instrument (Luminex Corporation, Austin, TX, USA).

### Analysis of trophic factor secretion

For evaluation of trophic factor secretion, BM-MSCs MSCs from 11 unrelated donors (5 female, 6 male; age: 50.9 y ± 13.3 y) (P2) were seeded to six-well plates at a subconfluent density of 20,000/cm^2^. The cells were kept under standard culture conditions and the medium was renewed after 24 hours to a precise volume of 3.0 ml per well. After 72 additional hours, culture supernatants were transferred to Eppendorf cups and frozen at −80°C for subsequent cytokine analysis.

Samples were sent to Multimetrix (Heidelberg, Germany) for multiplex analysis of HGF, LIF, VEGF-A, bFGF and NGFB expression in a commercial Luminex® system. Expression of BMP4 and Angiopoietin-1 was analyzed with Quantikine™ ELISA systems (R&D Systems) per the manufacturer’s instructions. ELISA standard curves were aligned using a five-parameter logistic regression model for sample quantification. Background concentration of all factors was measured in complete culture medium and subtracted from the detected concentrations of the respective culture supernatants for both Luminex® analysis and ELISAs. All samples were analyzed in technical duplicates.

### Analysis of hormone and growth factor receptor expression

For analysis of androgen receptor and FGFR3 expression, BM-MSCs from 14 unrelated donors (7 female, 7 male; age: 53.7 y ± 13.8 y) (P2) were seeded into 12-well plates in a density of 20,000 cells/cm^2^ and kept under standard culture conditions for 24 hours. Thereafter, the cell monolayers were washed with ice cold PBS, lysed in 300 μl Procarta™ lysis buffer (Affymetrix, Santa Clara, CA, USA) and frozen at −80°C.

Samples were analyzed for FGFR3 and androgen receptor content with ELISA systems (USCN Life Science, Wuhan, China) per the manufacturer’s instructions. ELISA standard curves were aligned using a five-parameter logistic regression model for sample quantification. Background concentration was measured in pure lysis buffer and subtracted from the detected concentrations of the respective cell lysates. All samples were analyzed in technical duplicates.

### Telomerase activity assay

To quantify telomerase activity, the Telo TAGGG Telomerase PCR ELISA kit (Roche Applied Science, Mannheim, Germany), based on the Telomeric Repeat Amplification Protocol (TRAP), was used on 2 × 10^5^ BM-MSCs at P1 according to the manufacturer’s instructions. Samples were classified as telomerase negative when the extinction value was <0.2 after subtracting the negative control.

### Statistical analysis

The statistical analyses were performed using ANOVA analysis of variance, the Spearman two-tailed correlation test, or the two-tailed Student’s *t*-test. Differences were considered significant when *P* <0.05.

## Results

### BM-MSC preparations are composed of BM-MSC subpopulations with a heterogenic phenotype

**Figure 1:**
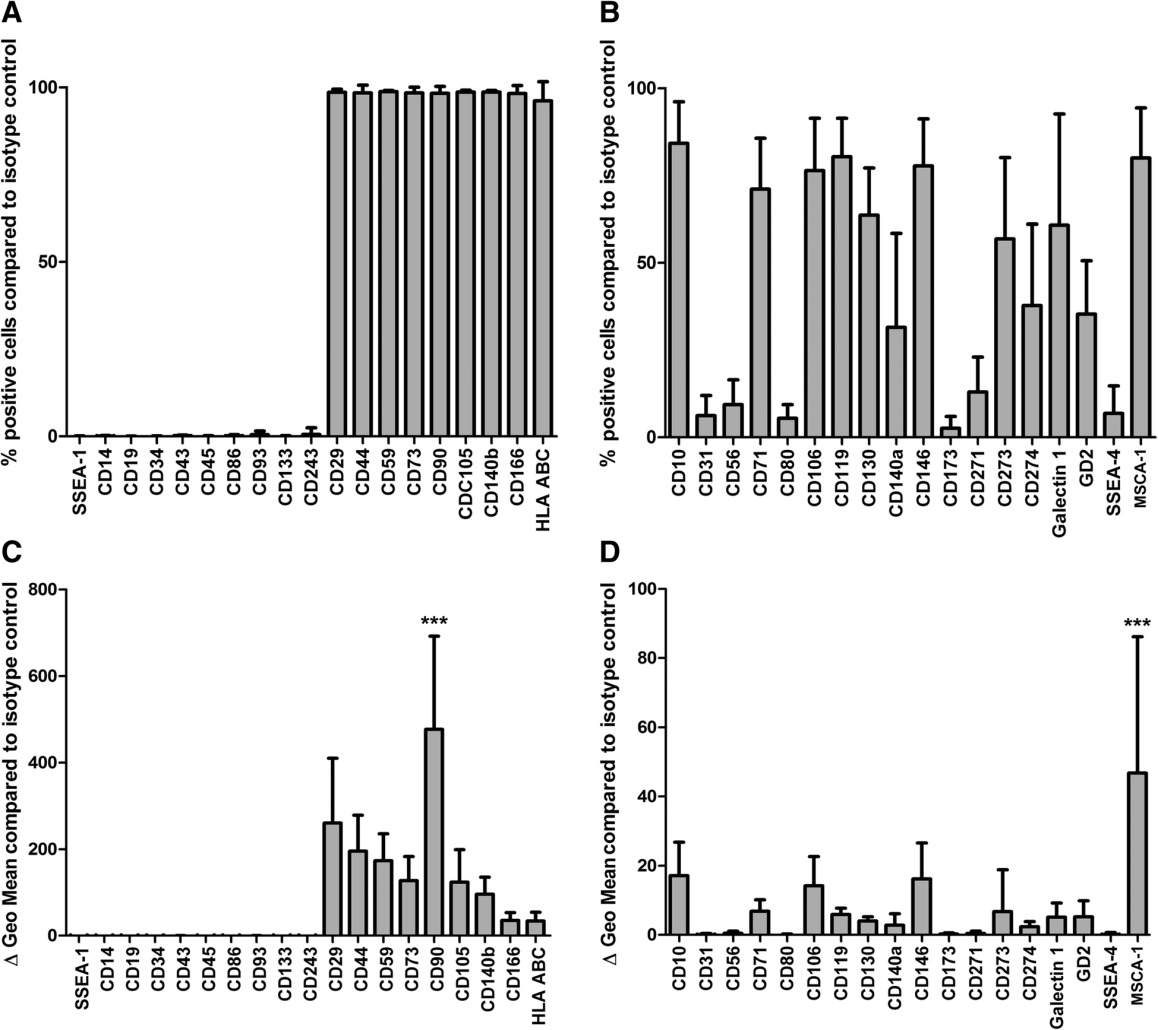
**Flow cytometric analyses.** The expression of CD10, CD14, CD19, CD29, CD31, CD34, CD43, CD44, CD45, CD56, CD59, CD71, CD73, CD80, CD86, CD90, CD93, CD105, CD106, CD119, CD130, CD133, CD140a, CD140b, CD146, CD173, CD166, CD243, CD271, CD273, CD274, Galectin 1, GD2, MSCA-1, SSEA-1, SSEA-4 and HLA class I was analyzed by flow cytometry in MSC preparations from multiple donors identifying three groups of marker expression. Markers that were expressed on all/most of the cells or on none/very few of the cells within the respective MSC preparation (**A**) and markers that identified MSC subpopulations by presence or absence of the respective marker (**B**) (n = 33 except for CD80 (n = 30), CD86 (n = 30), CD119 (n = 27), CD130 (n = 34), CD273 (n = 30), CD274 (n = 30), Galectin 1 (n = 24), and SSEA-4 (n = 36)). Analyses of specific antibody mediated fluorescence per cell (ΔGeo Mean) identified markers that were expressed at high and low density per cell (**C**, **D**) (n = 36 except for CD80 (n = 32), CD86 (n = 32), CD119 (n = 29), CD130 (n = 37), CD273 (n = 32), CD274 (n = 32), Galectin 1 (n = 26), and SSEA-4 (n = 39)). ANOVA analysis of variance followed by Tukey’s Multiple Comparison Test (***P <0.001). Error bars: SD.

Flow cytometric analysis was performed with 38 BM-MSC preparations (even distribution of gender and age (20 male, age: 54.4 y ± 11.3 y SD; 18 female, age 50.3 y ± 16.9 y SD)) revealing three different patterns of surface markers on the cells that composed the BM-MSC preparations *in vitro*: First, CD29, CD44, CD59, CD73, CD90, CD105, CD140b, CD166 and HLA ABC were expressed on the majority of cells within the MSC preparations (>96%) (n = 33). Second, no or very few (≤0.5%) cells expressing CD14, CD19, CD34, CD43, CD45, CD86, CD93, CD133, CD243 and SSEA-1 could be detected in the BM-MSC preparations (n = 33 except for CD86 (n = 30)) (Figure 1A). In contrast to these two marker groups, a third marker group showed a great heterogeneity of BM-MSC subpopulations expressing these antigens (2.6% to 84.2%) within the respective BM-MSC preparations with great standard variations between the BM-MSC preparations: CD10 (84.2% ± 11.7% SD), CD31 (6.2% ± 5.7% SD), CD56 (9.4%±7.0%SD), CD71 (71.0% ± 14.4% SD), CD80 (5.5% ± 3.8% SD), CD106 (76.4% ± 14.8% SD), CD119 (80.3% ± 10.9% SD), CD130 (63.6% ± 13.3% SD), CD140a (31.5% ± 26.5% SD), CD146 (77.7% ± 13.3% SD), CD173 (2.6% ± 3.3% SD), CD271 (13.0% ± 9.8% SD), CD273 (56.9% ± 22.8% SD), CD274 (37.8% ± 22.9% SD), Galectin 1 (60.8% ± 31.2% SD), GD2 (35.3% ± 15.1% SD), MSCA-1 (80.0% ± 14.1% SD), and SSEA-4 (6.8% ± 7.8% SD) (n = 33 except for CD80 (n = 30), CD119 (n = 27), CD130 (n = 34), CD273 (n = 30), CD274 (n = 30), Galectin 1 (n = 24), and SSEA-4 (n = 36)) (Figure 1B).

Analyses of specific antibody mediated mean fluorescence intensity (MFI) per cell (ΔGeo Mean) showed that the markers that were expressed on most of the cells (Figure 1A) were also expressed at high density per cell (Figure 1C). Here, CD90 could be identified as the marker that was expressed at highest density per cell (476.8 MFI ±212 SD). In the group of markers that showed a heterogenic expression profile MSCA-1 was expressed at highest density per cell (46.7 MFI ±38.8 SD) (n = 36 except for CD80 (n = 32), CD86 (n = 32), CD119 (n = 29), CD130 (n = 37), CD273 (n = 32), CD274 (n = 32), Galectin 1 (n = 26), and SSEA-4 (n = 39)) (Figure 1D).

### The phenotype of BM-MSCs from younger donors is different compared to older donors

**Figure 2:**
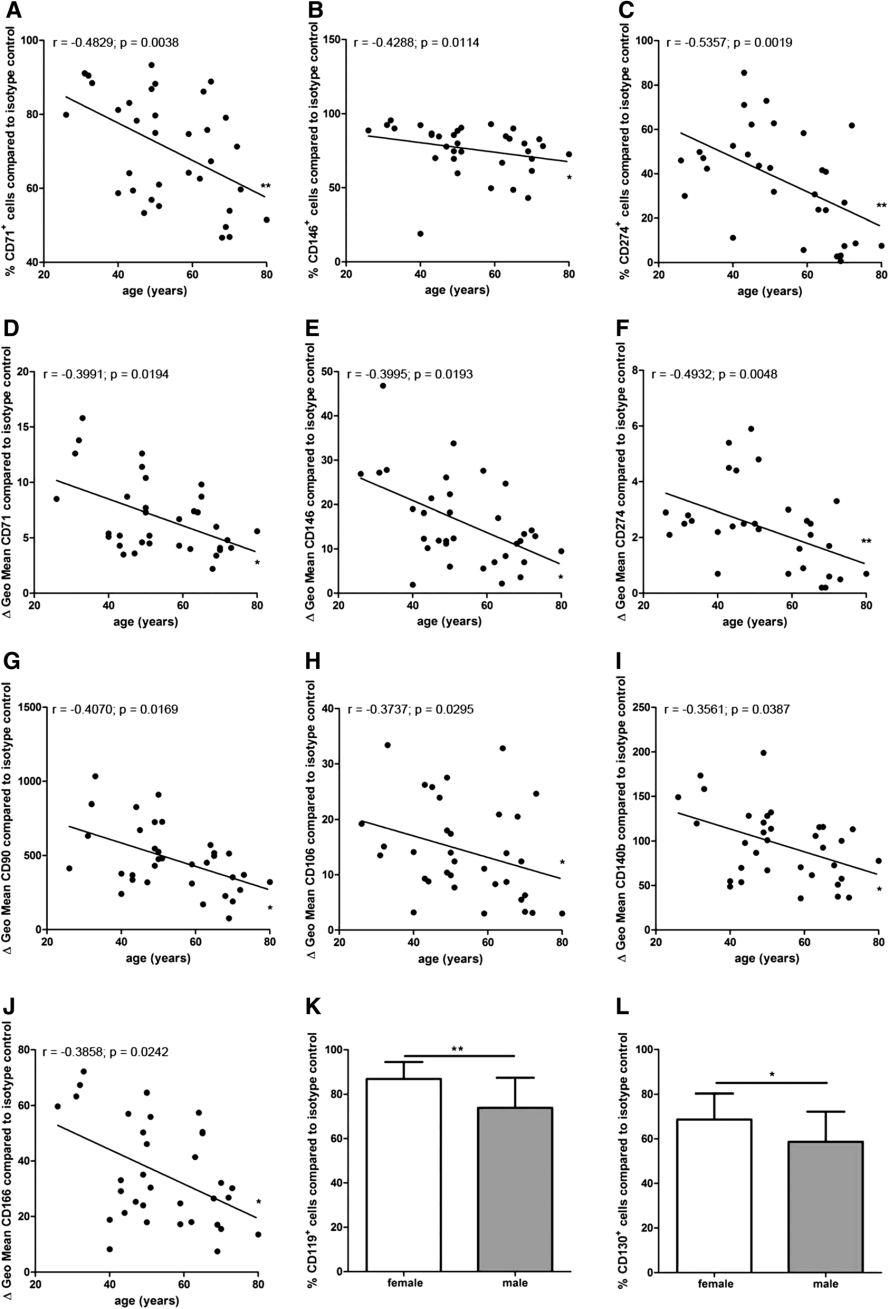
**Correlation analyses of marker expression to donor age and gender distribution.** The percentage of CD71^+^, CD146^+^, and CD274^+^ cells and the specific antibody mediated fluorescence per cell (ΔGeo Mean) of CD71, CD90, CD106, CD140b, CD146, CD166 and CD274 correlated negatively with the donor age (n = 34 except for CD274 (n = 31)) (**A**-**J**). Spearman two-tailed correlation test (*P <0.05; **P <0.01). MSC preparations from female donors harbored significantly more CD119^+^ cells (n = 11) and CD130^+^ cells (n = 16) compared to MSC preparations from male donors (CD119^+^ cells: n = 17; CD130^+^ cells: n = 19) (**K**, **L**). Two-tailed Student’s *t*-test (*P <0.05; **P <0.01). Error bars: SD.

The percentage of CD71^+^, CD146^+^, and CD274^+^ cells correlated negatively with the donor age indicating that BM-MSC preparations from younger donors contained more CD71^+^, CD146^+^, and CD274^+^ MSC subpopulations compared to BM-MSC preparations from older donors (n = 34 except for CD274 (n = 31)) (Figure 2A-C).

Similarly, the specific antibody mediated fluorescence per cell (ΔGeo Mean) of CD71, CD90, CD106, CD140b, CD146, CD166 and CD274 correlated negatively with the donor age indicating that the expression of CD71, CD90, CD106, CD140b, CD146, CD166 and CD274 per cell was higher in BM-MSC preparations from younger donors compared to MSC preparations from older donors (n = 34 except for CD274 (n = 31)) (Figure 2D-J).

### BM-MSC preparations from female donors contain more CD119^+^ and CD130^+^ cells and smaller cells that divide more rapidly

**Figure 3:**
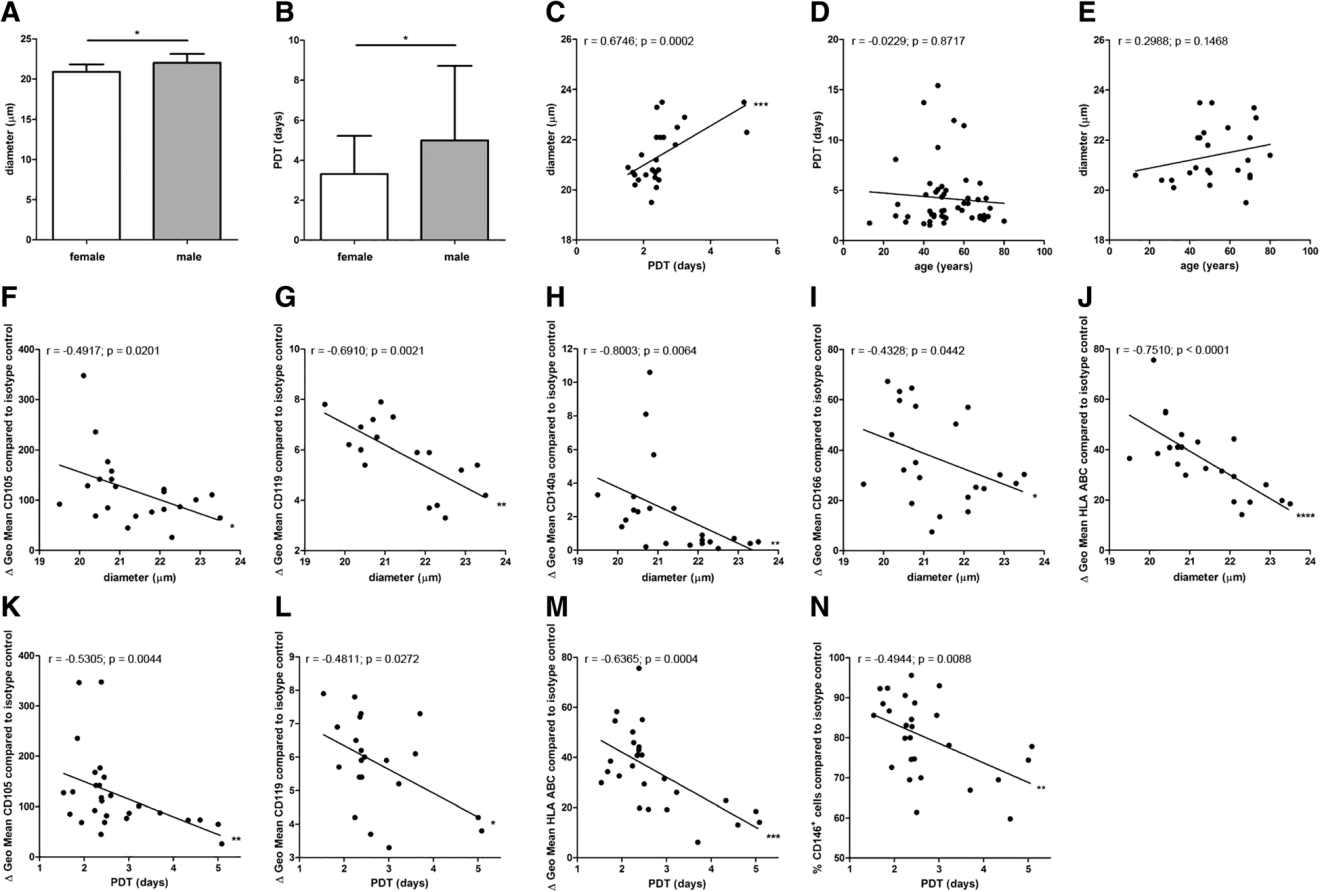
**Analyses of cell size and growth kinetics.** The diameter of MSCs from female donors (n = 14) was slightly but significantly smaller compared to male donors (n = 11) (**A**). The PDT of MSCs from female donors (n = 25) was significantly reduced compared to MSCs from male donors (n = 27) (**B**). Two-tailed Student’s *t*-test (*P <0.05). Error bars: SD. The cell diameter correlated positively to the PDT (n = 25) (**C**). No correlation was found between the donor age and the proliferation capacity of the MSCs (n = 52) (**D**) and between the donor age to the cell size (n = 25) (**E**). The specific antibody mediated fluorescence per cell (ΔGeo Mean) of CD105, CD119, CD140a, CD166 and HLA ABC correlated negatively with the cell size (n = 22 except for CD119 (n = 17)) (**F**-**J**). The specific antibody mediated fluorescence per cell (ΔGeo Mean) of CD105, CD119, and HLA ABC correlated negatively with the PDT (n = 27 except for CD119 (n = 21)) (**K**-**M**). The PDT correlated negatively with the percentage of CD146^+^ cells within the MSC preparations (n = 27) (**N**). Spearman two-tailed correlation test (*P <0.05; **P <0.01; ***P <0.001; ****P <0.0001).

BM-MSC preparations from female donors harbored significantly more CD119^+^ cells (86.8% ± 7.3% SD) (n = 11) and CD130^+^ cells (68.6% ± 11.3% SD) (n = 16) compared to BM-MSC preparations from male donors (CD119^+^ cells: 73.9% ± 13.1% SD (n = 17); CD130^+^ cells: 58.7% ± 13.2% SD (n = 19)) (Figure 2K,L). The diameter of BM-MSCs from female donors (20.9 μm ± 0.8 μm SD) (n = 14) was slightly but significantly smaller compared to male donors (22.0 μm ± 1.1 μm SD) (n = 11) (Figure 3A). The population doubling time (PDT) of BM-MSCs from female donors (3.3 d ± 1.9 d SD) (n = 25) was significantly reduced compared to BM-MSCs from male donors (5.0 d ± 3.7 d SD) (n = 27) (Figure 3B) indicating the presence of smaller, more rapidly dividing cells in BM-MSC preparations from female donors compared to male donors. This finding was confirmed by the positive correlation of the cell size to the PDT (n = 25) (Figure 3C). No correlation was found between the donor age and the proliferation capacity of the BM-MSCs (n = 52) (Figure 3D) and between the donor age to the cell size (n = 25) (Figure 3E).

### BM-MSC preparations containing smaller cells are composed of subpopulations that express surface markers at a higher density

The specific antibody mediated fluorescence per cell (ΔGeo Mean) of CD105, CD119, CD140a, CD166 and HLA ABC correlated negatively with the cell size indicating that the expression of these antigens was higher on cells within BM-MSC preparations that contain more small cells (n = 22 except for CD119 (n = 17)) (Figure 3F-J).

### BM-MSC preparations containing more rapidly dividing cells are composed of subpopulations that express surface markers at a higher density

The specific antibody mediated fluorescence per cell (ΔGeo Mean) of CD105, CD119 and HLA ABC correlated negatively with the PDT indicating that the expression of these antigens was higher on cells within BM-MSC preparations that contain more rapidly dividing cells (n = 27 except for CD119 (n = 21)) (Figure 3K-M). Moreover, the PDT correlated negatively with the percentage of CD146^+^ cells within the BM-MSC preparations (n = 27) (Figure 3N).

### High-clonogenic BM-MSCs are smaller, divide more rapidly and are more frequent in BM-MSC preparations from younger, female donors

**Figure 4:**
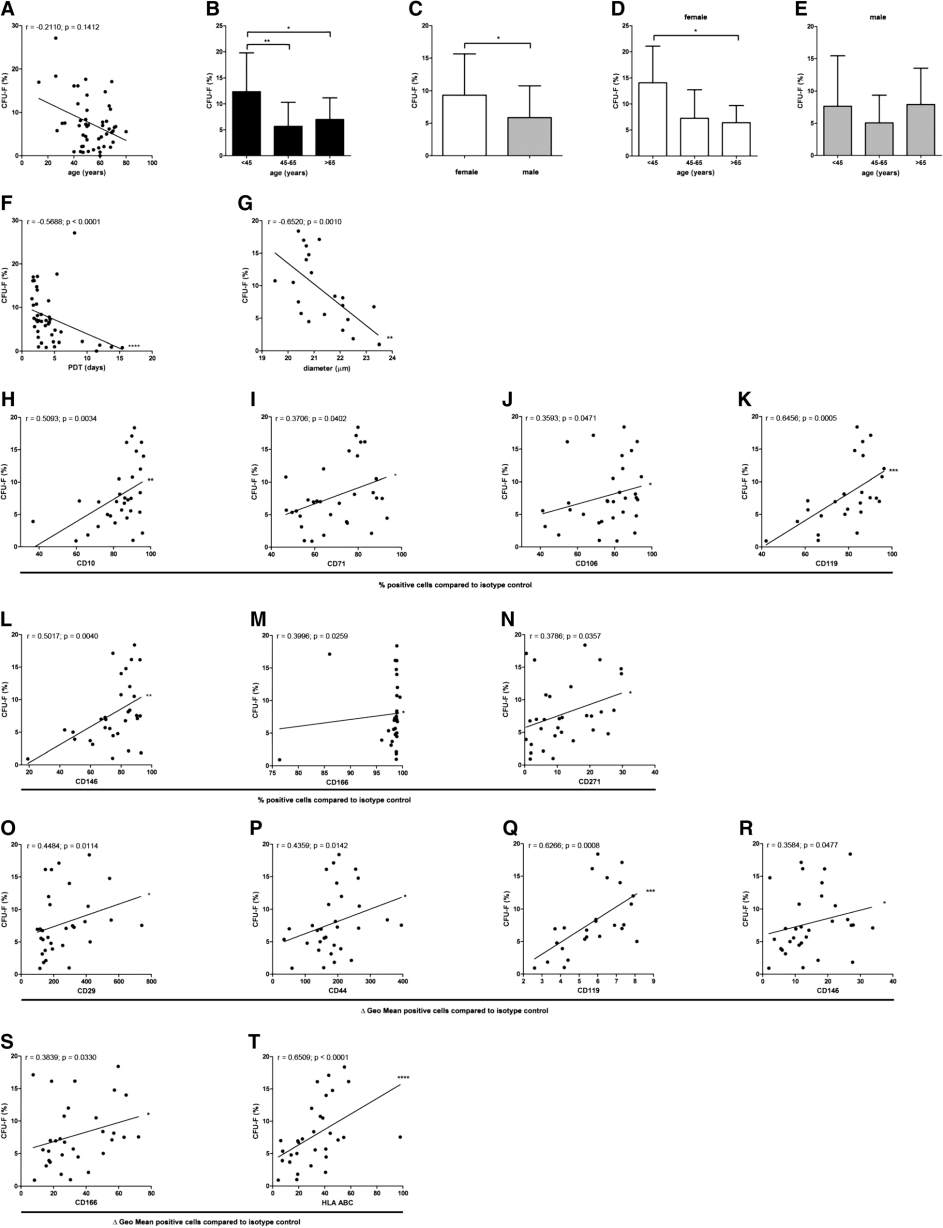
**Analyses of clonogenic potential.** Comparing all ages and both genders, MSC preparations from the BM of younger donors trended toward more colony forming cells than MSC preparations from older donors (n = 50) (**A**). Spearman two-tailed correlation test. Comparing age groups including both genders, significantly more colonies could be detected in young donors (<45 years) (n = 11) compared to middle-aged (n = 26) and older donors (n = 13) (**B**). ANOVA analysis of variance followed by Tukey’s Multiple Comparison Test (*P <0.05; **P <0.01). Error bars: SD. In BM-MSC preparations from female donors more colony forming cells could be detected (n = 23) compared to BM-MSC preparations from male donors (n = 27) (**C**). Two-tailed Student’s *t*-test (*P <0.05). Error bars: SD. Age-group specific analyses for each gender confirmed that more colonies could be detected in young female donors (<45 years) compared to older donors (**D**, **E**). ANOVA analysis of variance followed by Tukey’s Multiple Comparison Test (*P <0.05). Error bars: SD. The clonogenic potential of the cells within the BM-MSC preparations was negatively correlated to the population doubling time (n = 42) and the cell diameter (n = 22) (**F**, **G**). Spearman two-tailed correlation test (**P <0.01; ****P <0.0001). The percentage of CD10^+^, CD71^+^, CD106^+^, CD119^+^ CD146^+^, CD166^+^ and CD271^+^ cells correlated positively with the clonogenic potential of the BM-MSCs (n = 31 except for CD119 (n = 25)) (**H**-**N**). The specific antibody mediated fluorescence per cell (ΔGeo Mean) of CD29, CD44, CD119, CD146, CD166 and HLA ABC correlated positively with the clonogenic potential of the MSCs (n = 31 except for CD119 (n = 25)) (**O**-**T**). Spearman two-tailed correlation test (*P <0.05; **P <0.01; ***P <0.001; ****P <0.0001).

To assess the clonogenic activity of the BM-MSC preparations, a total of 50 BM donors were analyzed (donor distribution shown in Additional file 3: Table S1). Comparing all ages and both genders, MSC preparations from the BM of younger donors trended toward more colony forming cells than MSC preparations from older donors (n = 50) (Figure 4A). Comparing age groups including both genders, significantly more colonies could be detected in young (<45 years) donors (12.3% ± 7.1% SD; n = 11) compared to middle-aged (45 to 65 years; 5.6% ± 4.5% SD; n = 26) and older donors (>65 years; 6.9% ± 4.0% SD; n = 13) (Figure 4B). In BM-MSC preparations from female donors more colony forming cells could be detected (9.3% ± 6.2% SD; n = 23) compared to BM-MSC preparations from male donors (5.9% ± 4.8% SD) (n = 27) (Figure 4C). Age group specific analyses for each gender confirmed that more colonies could be detected in young female donors (<45 years) compared to older donors (Figure 4D,E). The clonogenic potential of the cells within the BM-MSC preparations was inversely related to the population doubling time (n = 42) and the cell diameter (n = 22) indicating a more rapid growth of smaller, high-clonogenic BM-MSC subpopulation(s) (Figure 4F,G).

The percentage of CD10^+^, CD71^+^, CD106^+^, CD119^+^ CD146^+^, CD166^+^ and CD271^+^ cells correlated positively with the clonogenic potential of the BM-MSCs (n = 31 except for CD119 (n = 25)) (Figure 4H-N). The specific antibody mediated fluorescence per cell (ΔGeo Mean) of CD29, CD44, CD119, CD146, CD166 and HLA ABC correlated positively with the clonogenic potential of the MSCs (n = 31 except for CD119 (n = 25)) (Figure 4O-T). These data suggest that CD10, CD29, CD44, CD71, CD106, CD119, CD146, CD166, CD271 and HLA ABC indicate BM-MSC preparations with high clonogenic potential.

The pooled human serum (PHS) used in our study was obtained from male donors only, consequently containing more testosterone (16.18 nmol/l) than would be expected in PHS from female individuals (normal range of testosterone in male serum: 9 to 30 nmol/l; normal range of testosterone in female serum: 0.5 to 2.6 nmol/l). Moreover, the PHS contained 75.12 pg/ml basic fibroblast growth factor (bFGF). To investigate possible contributions of testosterone and/or bFGF to the observed differences between female and male BM-MSCs, we compared androgen receptor and fibroblast growth factor receptor 3 (FGFR3) expression of BM-MSC preparations by ELISA. No difference was detected in the androgen receptor or FGFR3 expression nor was there any significant correlation of these receptors to donor age (n = 14 (7 female, 7 male)) (see Additional file 4: Figure S1).

### BM-MSC preparations from female donors demonstrated higher levels of allogeneic T cell proliferation suppression

**Figure 5:**
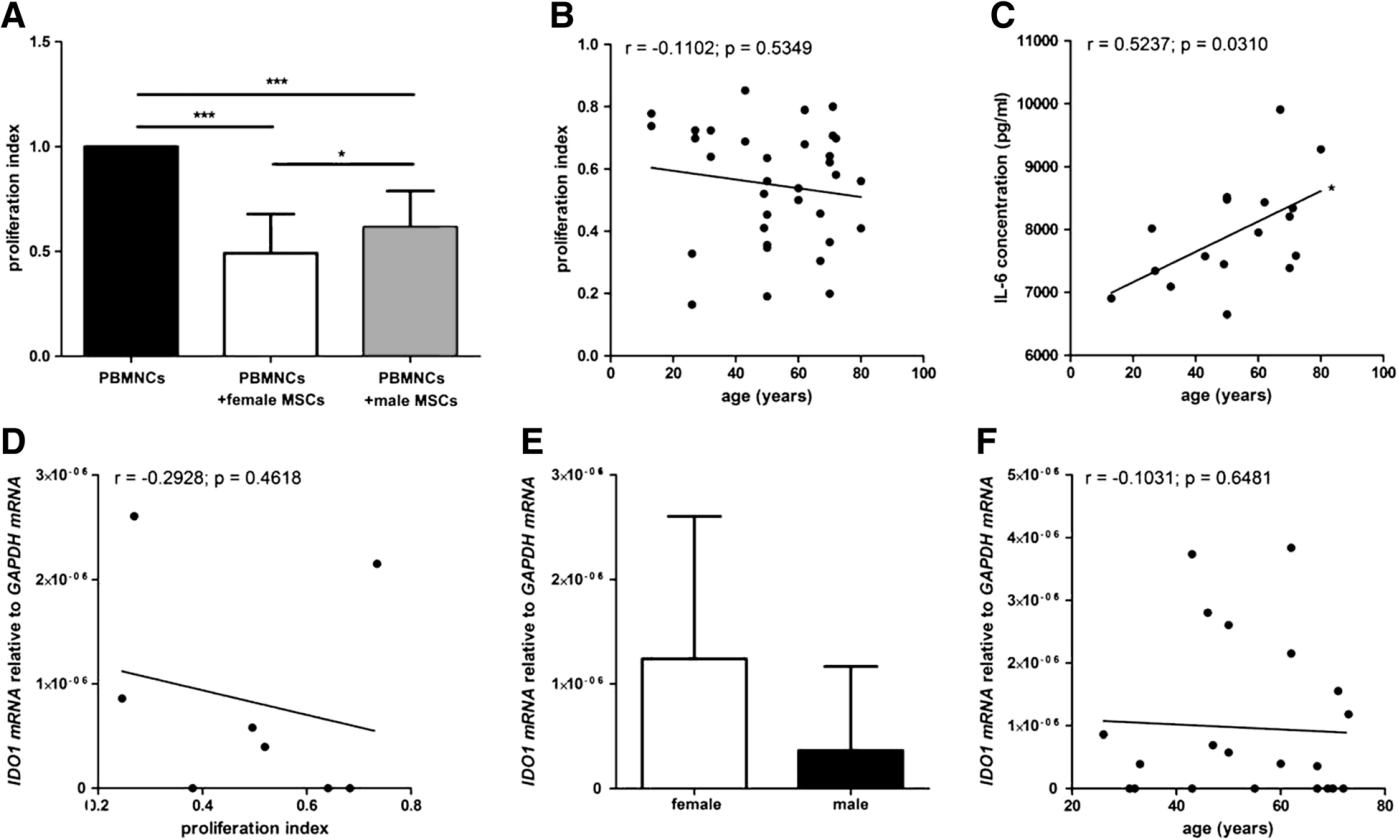
**Analyses of the immunosuppressive potential of MSCs.** MSCs from both female and male donors significantly suppressed the proliferation of activated allogeneic T cells. MSCs from female donors (n = 18) showed a significantly higher potency to suppress T cell proliferation than MSCs from male donors (n = 16). Data are normalized to the proliferation index of PBMNCs alone (**A**). ANOVA analysis of variance followed by Tukey’s Multiple Comparison Test (*P <0.05; ***P <0.001). Error bars: SD. No correlation of the donor age to the potential to suppress T cell proliferation could be detected (**B**). Upon co-culturing of MSCs from older donors with activated allogeneic T cells more IL-6 could be detected in the supernatant compared to MSCs from younger donors that were co-cultured with activated T cells (n = 17) (**C**). Spearman two-tailed correlation test (*P <0.05). Although a trend toward a negative correlation was observed, no significant correlation of *IDO 1* mRNA expression to T cell proliferation could be detected (n = 8) (**D**). Spearman two-tailed correlation test. No difference of *IDO 1* mRNA expression from male MSC donors compared to female MSC donors could be detected (n = 22) (**E**). Two-tailed Student’s *t*-test. Error bars: SD. No correlation of the *IDO 1* mRNA expression to the donor age could be observed (n = 22) (**F**). Spearman two-tailed correlation test.

BM-MSCs from both female and male donors significantly suppressed the proliferation of activated allogeneic T cells obtained from six different donors. BM-MSCs from female donors (nine donors, tested with allogeneic T cells from two different donors; n = 18) showed a significantly increased suppression of T cell proliferation compared to BM-MSCs from male donors (eight donors, tested with allogeneic T cells from two different donors; n = 16) (Figure 5A). No correlation of the donor age to the potential to suppress T cell proliferation could be detected (Figure 5B). Upon co-culturing of BM-MSCs from older donors with activated allogeneic T cells more interleukin IL-6 could be detected in the supernatant compared to BM-MSCs from younger donors that were co-cultured with activated T cells (n = 17) (Figure 5C). No differences could be detected with respect to IL-2, IL-4, IL-8, IL-10, IFN-γ and TNF-α concentrations in the supernatants of BM-MSCs cultured with or without activated allogeneic T cells (data not shown).

BM-MSCs from both female and male donors expressed *IDO 1* mRNA but no *IDO 2* mRNA (data not shown). Although a trend toward a negative correlation was observed, no significant correlation of *IDO 1* mRNA expression to T cell proliferation could be detected (n = 8) (Figure 5D). No difference of *IDO 1* mRNA expression from male BM-MSC donors compared to female BM-MSC donors could be detected and no correlation of the *IDO 1* mRNA expression to the donor age could be observed (n = 22) (Figure 5E,F).

### The *in vitro* mesodermal differentiation capacity of BM-MSCs is not affected by donor age and donor gender but by phenotype

**Figure 6:**
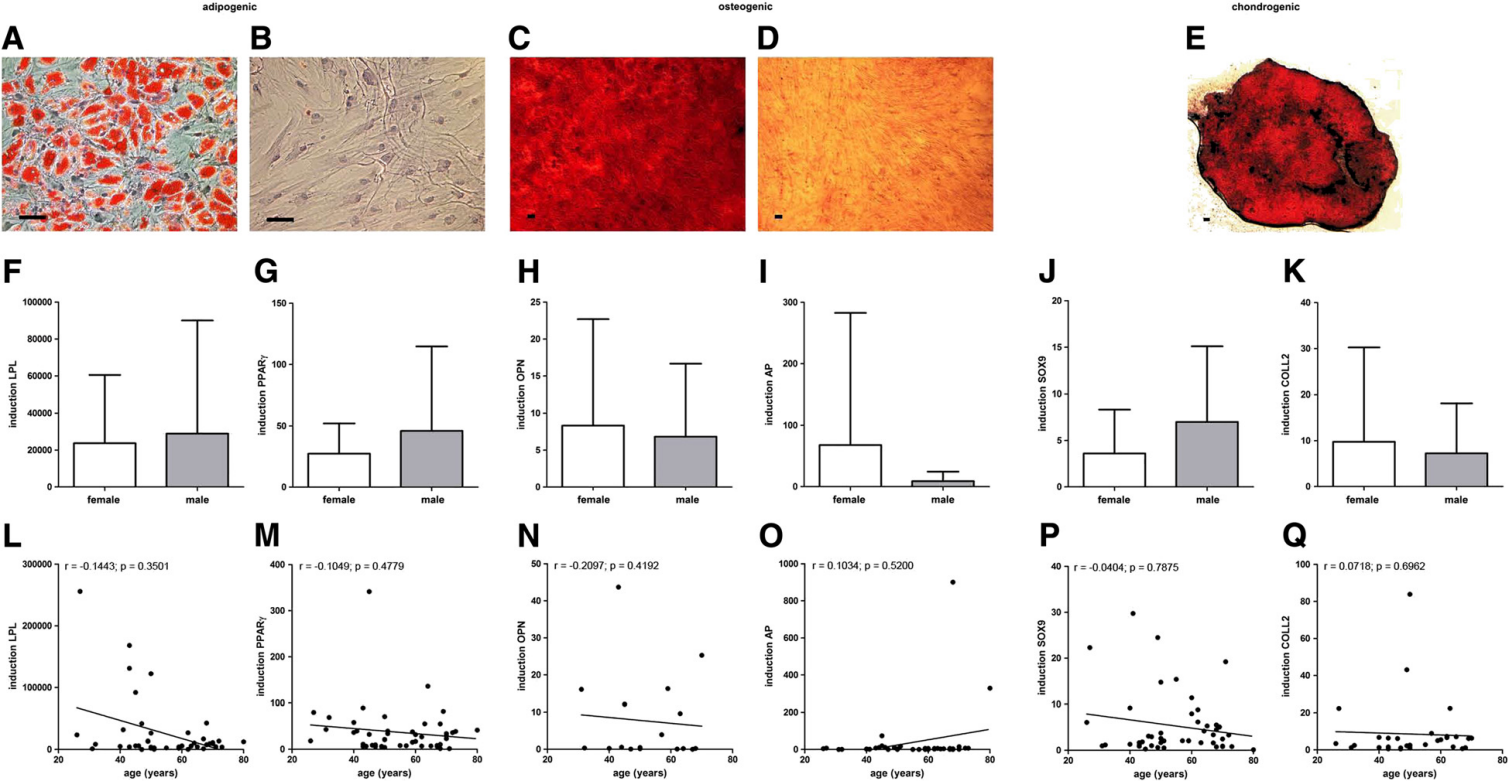
**Mesodermal differentiation potential of MSCs.** No donor age or gender related differences could be detected for the adipogenic, osteogenic and chondrogenic *in vitro* differentiation capacity of the MSC preparations as analysed by lineage specific staining (Oil Red O for adipogensis; Alizarin Red for osteogenesis, and Safranain O for chondrogenesis (n = 40); differentiation: **A**, **C**, **E**; negative controls: **B**, **D**) and no statistically significant differences could be detected for the lineage specific mRNA expression (*LPL* (n = 44) and *PPARγ* (n = 48) for adipogenesis; *OPN* (n = 17) and *AP* (n = 41) for osteogenesis; *SOX9* (n = 47) and *COLL2* (n = 32) for chondrogenesis; **F**-**Q**). Two-tailed Student’s *t*-test and Spearman two-tailed correlation test. Error bars: SD. Scale bars: 100 μm.

No donor age or gender related differences could be detected for the adipogenic, osteogenic and chondrogenic *in vitro* differentiation capacity of the BM-MSC preparations as analyzed by lineage specific staining (Oil Red O for adipogenesis; Alizarin Red for osteogenesis, and Safranain O for chondrogenesis (n = 32); Figure 6A-E) and no statistically significant differences could be detected for the lineage specific mRNA induction (*LPL* (n = 44) and *PPARγ* (n = 48) for adipogenesis; *OPN* (n = 17) and *AP* (n = 41) for osteogenesis; *SOX9* (n = 47) and *COLL2* (n = 32) for chondrogenesis; Figure 6F-Q).

The percentage of CD10^+^ cells (n = 27) and the specific antibody mediated fluorescence per cell (ΔGeo Mean) of CD119 (n = 22) correlated positively with the induction of *PPARγ* mRNA under adipogenic differentiation (Additional file 5: Figure S2A-D). Moreover, the percentage of GD2^+^ cells and the specific antibody mediated fluorescence per cell (ΔGeo Mean) of GD2 correlated negatively with the induction of *LPL* mRNA under adipogenic differentiation (n = 26) (Additional file 5: Figure S2E,F).

### The phenotype of BM-MSCs but not donor age or gender affects the secretion of trophic factors

BM-MSCs secreted the highest concentrations of VEGF-A and HGF, followed by LIF, Angiopoietin-1, bFGF and NGFB. BMP4 could not be detected in the supernatants of the tested BM-MSC preparations (six female, five male; age: 50.9 y ± 12.7 y SD) (see Additional file 6: Figure S3A). Correlation analyses of the six secreted factors to markers potentially defining BM-MSC subpopulations (Figure 1) revealed a significant negative correlation of HGF secretion to the expression of CD71, CD140b and Galectin 1 (n = 11 except for Galectin 1 (n = 9)) (see Additional file 6: Figure S3B), and no positive correlation of the tested markers to the secretion of trophic factors could be identified. Moreover, neither donor age nor gender affected the secretion of trophic factors (see Additional file 6: Figure S3C,D). Interestingly, we detected Angiopoietin-1 in the supernatants of only 4 of the 11 tested BM-MSC populations (see Additional file 7: Table S2). Age and gender distribution within the “secretor” group (53.3 y ± 16.6 y; two female, two male) and the “non-secretor” group (49.6 y ± 9.6 y; three female, four male) was balanced, and no correlation of the marker expression to the Angiopoietin-1 “(non-)secretor” status of the BM-MSCs could be identified (see Additional file 6: Figure S3E,F).

### *Oct4, Nanog* and *Prdm14* mRNA expression is low compared to pluripotent stem cells and is not affected by age or gender in BM-MSC preparations *in vitro*

**Figure 7:**
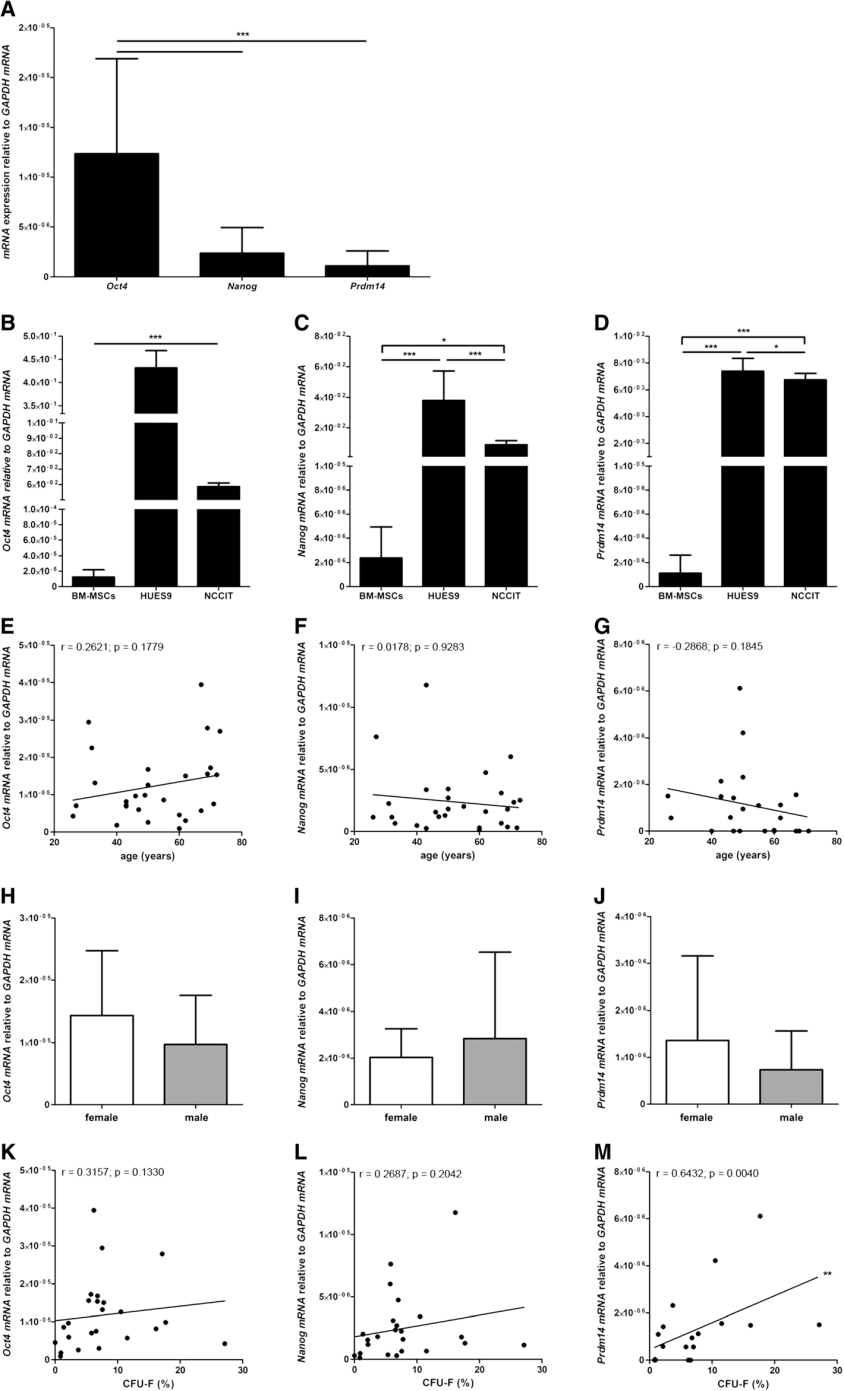
**Expression of *Oct4*, *Nanog *and *Prdm14 *mRNA and functional relevance.** The MSCs expressed significantly more *Oct4* mRNA than *Nanog* or *Prdm14* mRNA; however, no significant difference in *Nanog* mRNA expression compared to *Prdm14* mRNA expression could be detected (n = 28 except for *Prdm14* (n = 23)) (**A**). Compared to pluripotent HUES9 cells and pluripotent NCCIT cells the expression of *Oct4* mRNA, *Nanog* mRNA and *Prdm14* mRNA was much lower in MSCs (**B**-**D**). Compared to NCCIT cells the HUES9 cells expressed more *Oct4* mRNA, *Nanog* mRNA and *Prdm14* mRNA (B-D). ANOVA analysis of variance followed by Tukey’s Multiple Comparison Test (*P <0.05; ***P <0.001). Error bars: SD. No correlation of the expression of *Oct4, Nanog* and *Prdm14* mRNA to donor age (n = 28 except for *Prdm14* (n = 23)) (**E**-**G**) or any gender-related differences could be detected (n = 28 except for *Prdm14* (n = 23)) (**H**-**J**). The clonogenic potential of MSCs correlated positively to the expression of *Prdm14* mRNA (n = 18) but not to *Oct4* (n = 24) or *Nanog* mRNA (n = 24) (K-M). Two-tailed Student’s *t*-test and Spearman two-tailed correlation test (**P <0.01). Error bars: SD.

*Oct4, Nanog* and *Prdm14* mRNA was expressed by BM-MSCs *in vitro* whereas *SOX2* mRNA could only be detected at minimal level in 5 out of 18 analyzed BM-MSC preparations. The BM-MSCs expressed significantly more *Oct4* mRNA than *Nanog* mRNA or *Prdm14* mRNA however, no significant difference in *Nanog* mRNA expression compared to *Prdm14* mRNA expression could be detected (n = 28 except for *Prdm14* (n = 23)) (Figure 7A). Compared to pluripotent human ESCs (HUES9) and a human pluripotent germ cell tumor line (NCCIT) the expression of *Oct4* mRNA, *Nanog* mRNA and *Prdm14* mRNA was much lower in BM-MSCs. HUES9 cells and NCCIT cells expressed 10^3^ to 10^4^ times more *Oct4*, *Nanog* and *PRDM14* mRNA than BM-MSCs (Figure 7B-D). Moreover, no correlation of the expression of *Oct4, Nanog* and *Prdm14 mRNA* to donor age (n = 28 except for *Prdm14* (n = 23)) (Figure 7E-G) or any gender-related differences could be detected (n = 28 except for *Prdm14* (n = 23)) (Figure 7H-J).

### *Prdm14* mRNA expression is positively correlated to the clonogenic potential of MSCs *in vitro*

The clonogenic potential of BM-MSCs correlated positively to the expression of *Prdm14 mRNA* (n = 18) but not to *Oct4* (n = 24) or *Nanog mRNA* (n = 24) (Figure 7K-M). Interestingly, the clonogenic potential of the BM-MSC preparations did not correlate to the induction of lineage specific mRNA expression (*LPL*, *PPARγ*, *OPN*, *AP*, *SOX9* and *COLL2*) under differentiation *in vitro* (data not shown; summary in Table 1). Moreover, the expression of *Oct4, Nanog* and *Prdm14* mRNA in undifferentiated BM-MSCs did not correlate to the induction of lineage specific mRNA expression under differentiation of the respective MSC preparations *in vitro* (*LPL*, *PPARγ*, *OPN*, *AP*, *SOX9* and *COLL2*) except for a negative correlation of *SOX9* mRNA expression under chondrogenic differentiation to *Oct4* (n = 25) and *Prdm14* mRNA (n = 19) expression (data not shown; summary in Table 1).

**Table 1:** Not significantly correlated parameters

**Parameter 1**	**Parameter 2**	**n**
Donor age	Diameter	25
Donor age	PDT	52
Donor age	Secretion of IL-2, IL-4, IL-8, IL-10, TNFα, IFNγ(BM-MSCs alone)	17
Donor age	Expression of *Oct4* mRNA	28
Donor age	Expression of *Nanog* mRNA	28
Donor age	Expression of *Prdm14* mRNA	23
Donor gender	Antigen density per cell(ΔGeo Mean Fluorescence)	34
Donor gender	Secretion of IL-2, IL-4, IL-6, IL-8, IL-10, TNFα, IFNγ(BM-MSCs with stimulated allogeneic T cells)	17
Donor gender	Secretion of IL-2, IL-4, IL-6, IL-8, IL-10, TNFα, IFNγ(BM-MSCs alone or with stimulated allogeneic T cells)	17
CFU-F	Induction of *LPL*, *PPARγ*, *OPN*, *AP, SOX9* and *COLL2* mRNA (differentiation)	13 to 39
CFU-F	Expression of *Oct4* mRNA	24
CFU-F	Expression of *Nanog* mRNA	24
Expression *Oct4* mRNA (undifferentiated)	Induction of *LPL*, *PPARγ*, *OPN*, *AP* and *COLL2* mRNA (differentiation)	25
Expression *Nanog* mRNA (undifferentiated)	Induction of *LPL*, *PPARγ*, *OPN*, *AP, SOX9,* and *COLL2* mRNA (differentiation)	25
Expression *Prdm14* mRNA (undifferentiated)	Induction of *LPL*, *PPARγ*, *OPN*, *AP* and *COLL2* mRNA (differentiation)	19
Donor age, donor gender	Secretion of Angiopoietin-1, NGFB, LIF, bFGF, VEGF-A, HGF	11

### Human BM-MSCs show no telomerase activity *in vitro*

No telomerase activity could be detected in 24 analyzed BM-MSC preparations (11 female, 13 male; average age 53.7 years ± 12.7 years SD) (data not shown).

## Discussion

BM-MSC preparations *in vitro* are known to be a heterogenic mix of adherently growing cells sharing similar, that is, spindle shaped, morphology [25]. Studies have revealed that BM-MSC preparations *in vitro* are composed of poorly defined subpopulations, and that a minority of clonally expanded MSCs showed full “tri-lineage” (adipogenic, osteogenic and chondrogenic) differentiation potential [12,14,26,27], pointing toward functional differences of BM-MSC subpopulations. In addition to intra-individual heterogeneity, donor-related variations of differentiation potential and growth kinetics of MSC preparations point toward significant inter-individual heterogeneity [11,12]. Studies that identify *in vivo* stromal cell subpopulations are rare and focus on very few markers [24,28,29]. Attempts were, therefore, made to find distinct markers to identify the most “potent” MSC subpopulations for possible clinical use [13,15,23,30-32], resulting in greatly improved characterization of MSC preparations *in vitro.* Whether a distinct phenotype correlated with specific functional properties was addressed by sorting and analyzing few subpopulations but likely left many uncharacterized. As to heterogeneity, *in vitro* data are not conclusive due to study-to-study variations on BM-MSC performance (for example, differentiation potential) and reproducibility challenges. Therefore, it is difficult to understand the *in vivo* nature and composition of the BM stroma as well as the impact of *in vitro* selection and culture conditions on BM-MSC subpopulations. Analysis of sorted BM-MSC subpopulations, requiring thorough optimization of culture conditions for each subpopulation, is one option. We analyzed great numbers of human BM-MSC preparations to identify statistically robust correlations of phenotype, donor gender and age to functional properties.

We identified three surface marker patterns on adherent BM-MSCs *in vitro:* (1) markers uniquely expressed by all cells; (2) markers not expressed by any cells; and (3) markers heterogeneously expressed by subpopulations. Here we saw prominent inter-individual heterogeneity. With respect to distinct phenotypes of BM-MSC subpopulations, these markers appear most interesting and probably allow correlation of MSC preparations to potency. The great heterogeneity of MSC preparations still hampers the development of marker-based potency assays for MSCs, such as the well-established quantification of CD34^+^ cells in hematopoietic stem cell preparations.

Quantification of cells expressing (or lacking) these markers allowed us to distinguish markers expressed on rare (≤10%) subpopulations *versus* those on more frequent subpopulations.

To what extent, if any, adult BM-MSCs exhibit stem cell properties (that is, self-renewal and broad differentiation capacity) has been under debate for over a decade [33]. Despite difficulties in distinguishing self-renewal from proliferation or selection of immortalized clones, studies suggest MSCs might have self-renewal capacity [34]. Clonal growth is associated with self-renewal, regulated by factors such as LIF and BMPs [35,36]. However, determination of a stem cell character cannot be based on clonal growth alone because the differentiation potential of cell clones derived from BM is highly variable and only a minority of clones exhibit mesodermal “tri-lineage” differentiation capacity [26]. Regarding differentiation capacity, functional data on non-mesodermal or myogenic differentiation of human BM-MSCs are missing or controversial [10,19,37] and no study has reported on differentiation of adult human BM-MSCs, isolated by plastic adherence, into cells or tissue(s) of all three germ layers upon blastocyst transfer or teratoma formation *in vivo*. The surface antigen SSEA-4 and the transcription factors Oct4, Nanog and Prdm14 are regarded as stem cell markers [33,38,39]. SSEA-4^+^ MSCs [40,41], multipotent adult progenitor cells (MAPC), marrow-isolated adult multi-lineage inducible cells (MIAMI) and very small embryonic-like (VSEL) stem cells were shown to express stem cell markers, and could be differentiated into various mesodermal and non-mesodermal cell types [42-47]. We have also shown *in vivo* that adult human BM harbors distinct stromal cell entities expressing Oct4, Nanog and SSEA-4 on the protein/antigen level [48]. To put these observations into context with BM-MSCs *in vitro*, we analyzed clonogenic potential, mesodermal differentiation potential, expression of stem cell markers, and telomerase activity of the BM-MSC preparations. A variety of antigens, but not SSEA-4, were correlated to BM-MSC preparations with high clonogenic potential. Notably, the clonogenic potential of BM-MSCs did not correlate to differentiation potential or telomerase activity, and BM-MSCs expressed much lower levels of stem cell marker mRNA compared to pluripotent stem cells. Our data suggest that BM-MSC preparations isolated and cultured under standard conditions do not contain cells with stem cell properties, in possible contrast to MAPC, MIAMI or VSEL stem cells that require specialized isolation and culture conditions.

Our correlation analyses suggest the presence of BM-MSC phenotypes with either higher (CD10, CD119) or lower (GD2) adipogenic differentiation potential *in vitro*. Human BM harbors adipogenic stromal cells expressing CD10 but not GD2 *in vivo*[48]. We speculate that this cell type was, among others, positively selected by plastic adherence thereby being able to give rise to terminally differentiated adipocytes when exposed to the respective differentiation medium *in vitro*. To verify this hypothesis, we suggest future studies on sorted cells investigating the adipogenic differentiation potential of CD10^+^CD119^+^GD2^-^ and CD10^-^CD119^-^GD2^+^ BM-MSC subpopulations.

BM-MSC-mediated immunomodulation mainly comes as potent immunosuppression [18,49]. Therefore, BM-MSCs are used in the clinic for treatment of graft-*versus*-host disease (GvHD) [6,50] and multiple sclerosis (MS) [9,51,52], and clinical trials to treat type-1 diabetes (T1D) are underway. Here we investigated the expression of factors in BM-MSC preparations that are involved in BM-MSC immunomodulation. We show for the first time that human BM-MSCs express *IDO1* but not *IDO2*, consistent with the fact that IDO1 but not IDO2 is involved in tryptophan degradation [53]. François *et al.*[54] reported on the great variability of seven BM-MSC preparations to suppress T cell proliferation. Moreover, in analyzing 21 data points, they observed a positive correlation between IDO production by BM-MSCs and their potential to suppress T cell proliferation [54]. In our study, despite donor-to-donor variability, MSCs from female donors showed significantly increased suppression of T cell proliferation compared to male donors. Although we observed only trends in greater *IDO1* mRNA expression in female BM-MSCs and stronger suppression of T cell proliferation by BM-MSCs expressing more *IDO1* mRNA, we hypothesize that the superior immunosuppressive properties of female BM-MSCs might be mediated by IDO1.

Galectin 1, located either on the surface or in the cytoplasma of BM-MSCs, plays an important role in MSC-mediated suppression of T cell proliferation [55] and Galectin 1^+^ stromal cells contribute to the hematopoietic niche in the BM [56]. We detected Galectin 1, and for the first time on human BM-MSCs, PDL-1 and PDL-2 on approximately 50% of the cells within BM-MSC preparations, however, at relatively low densities per cell. Activation of Programmed Death 1 (PD-1) by the negative co-stimulatory molecules PDL-1 and PDL-2 leads to inactivation of T cells [57] and murine BM-MSCs were shown to exert their immunosuppressive function using the PD-1 pathway [58,59]. We did not observe significant correlations of function, in particular immunomodulation, to the expression of Galectin 1, PDL-1 or PDL-2 on BM-MSCs. However, our results do not negate potential contributions of these molecules in human BM-MSC function as the functional analyses of the immunomodulatory properties were performed with fewer BM-MSC preparations, possibly hampering the identification of significant differences or correlations.

Many functional parameters of BM-MSCs did not correlate to donor age or gender (Table 1). We, however, found that high-clonogenic BM-MSCs were smaller, divided more rapidly and were more frequent in BM-MSC preparations from younger, female donors. These findings correspond to a study that reported a higher growth rate and clonogenic potential of BM-MSCs from children *versus* adults [60]. Alves *et al.*[61] recently associated several *in vitro* characteristics of BM-MSCs to donor age. We found no correlation of donor age to adipogenic, osteogenic and chondrogenic differentiation *in vitro* as confirmed by an extended panel of lineage specific markers. We identified several differentially expressed proteins on BM-MSCs from younger donors compared to older donors. Interestingly, none of these targets correlated to donor age at the mRNA level in the Alves *et al.* study [61]. Besides possible age-related post-transcriptional impact on protein expression, the exposure of fetal bovine serum plus addition of bFGF in this study (*versus* PHS without any additional growth factors in our study) could have contributed to these discrepant results.

Studies have shown that MSC function *in vivo* is mediated by secreted trophic factors [7,62-66] although few studies, mainly on rodent MSCs, report gender dimorphism of MSC secretion of trophic factors or cytokines [67,68]. We propose the pre-transplantation assessment of BM-MSC-secreted trophic factors as a clinically relevant issue. We, therefore, analyzed the secretion of factors shown to mediate tissue regeneration and immunomodulation: VEGF-A (angiogenesis), HGF (angiogenesis, anti-proliferative effects on T-cells), LIF (induction of Foxp3^+^ T cells, anti-proliferative effects on T-cells), Angiopoietin-1 (angiogenesis), bFGF (angiogenesis, cell proliferation and migration), BMP4 (cell proliferation, osteogenesis) and NGFB (neuroprotection) [18,22]. We detected Angiopoietin-1 in the supernatants of only 4 of 11 tested BM-MSC preparations thereby identifying “Angiopoietin-1-secretor” and “Angiopoietin-1-non-secretor” BM-MSC preparations. Angiopoietin-1 secretor status was not determined by age, gender or marker expression. For further characterization we recommend RNA microarray screening analyses followed by functional assessments *in vitro* and *in vivo*. Previous studies reported on rat BM-MSC secretion of BMP4, a factor in proliferation and differentiation [69,70]. Interestingly, we could not detect BMP4 in the supernatants of human BM-MSC preparations, which suggests that secreted BMP4 might not be an ideal target to assess human BM-MSC preparations *in vitro.*

PDGF and bFGF play pivotal roles in MSC proliferation [71,72]. We detected CD140b (PDGFRb) on nearly all cells in the BM-MSC preparations. For CD140a (PDGFRa) expression, though possibly defining a subpopulation, we did not find a difference in gender distribution or correlation to BM-MSC proliferation. We, therefore, lack evidence of a crucial role for PDGF in gender dimorphism of BM-MSC proliferation. We then evaluated the possible influence of bFGF on BM-MSC proliferation. FGF receptors play important roles in proliferation, differentiation and possibly self-renewal of MSCs [73-75]. Kim *et al.*[73] showed that human synovium-derived MSCs transcribed *FGFR 1–4* mRNA but expressed only FGFR3 at the protein level. We analyzed FGFR3 protein expression in BM-MSCs without detecting differences between female and male donors, or identifying a correlation to donor age. The PHS used in our study was obtained from male donors only, with consequently more testosterone than expected from female individuals. To evaluate the role of testosterone in BM-MSC gender dimorphism, we analyzed the expression of androgen receptors in BM-MSCs. We found no difference between genders or a correlation to donor age. This corresponds to studies reporting that treatment with sex hormones (dihydrotestosterone, 17β-estradiol) did not increase the proliferative capacity of human MSCs [76,77].

Last, we identify three markers associated with several functional properties of BM-MSCs, that is, CD119, CD146 and HLA ABC (Table 2). We suggest future studies on sorted CD119^+^, CD146^+^ and HLA ABC^+^ BM-MSCs to analyze the expression profile of genes that mediate therapeutic potential as well as performance in functional assays.

**Table 2:** Assignment of functional and phenotypical properties to CD119, CD146 and HLA ABC expression

**Marker**	**Parameter**	**Correlation**	**n**
**CD119** (IFNγR1) antigen density per cell (ΔGeo Mean Fluorescence)	CFU-F	Positive	25
	diameter	Negative	17
	PDT	Negative	21
	Induction *PPARγ* mRNA	Positive	22
	Female donors > male donors*	n.a.	28
**CD146** (MCAM) antigen density per cell (ΔGeo Mean Fluorescence)	CFU-F	Positive	31
	PDT*	Negative	27
	Donor age	Negative	34
**HLA ABC** antigen density per cell (ΔGeo Mean Fluorescence)	CFU-F	Positive	31
	Diameter	Negative	22
	PDT	Negative	27

*Percentage of antigen positive cells.

## Conclusions

It has become evident that the currently most widely used BM-MSC isolation technology, that is, culture of plastic adherent BM-cells and removal of non-adherent BM-cells, is a significant selection process for BM-MSC subpopulations. Hereby, rare MSC subpopulations that possibly feature stem cell-related properties will be lost. On the other hand, a heterogenic mix of MSC subpopulations adapts very well to these conditions and proliferates *in vitro* as BM-MSC preparations. By analyzing and extensively characterizing a great number of BM-MSC preparations, we overcame a major challenge in research on primary BM-MSCs of donor-to-donor variation. We hereby identify phenotypes featuring functional properties that are partially donor-related, and propose future studies on CD119^+^, CD146^+^, HLA ABC^+^ BM-MSC subpopulations, as well as on Angiopoietin-1 secreting and non-secreting BM-MSC preparations.

For clinical production of BM-MSCs, markers that correlate positively and negatively to functional properties of BM-MSC preparations and markers that define rare subpopulations could be useful for the development of assays to define release or characterization criteria for quality control purposes and clinical applications. Moreover, the gender-related effect on growth kinetics of BM-MSCs could help to plan their scale-up production.

For clinical applications, our data might, on one hand, provide evidence to initiate clinical phase I trials comparing the immunomodulatory potential of allogeneic BM-MSC preparations from female to male donors for diseases such as GvHD, MS or T1D. On the other hand, donor age or gender might not affect BM-MSC performance in clinical applications where BM-MSC-derived trophic factors are considered to contribute substantially to efficacy of the cell therapy (for example, stroke or myocardial infarction).

## Abbreviations

(b)FGF(R): (basic) fibroblast growth factor (receptor); BM: Bone marrow; BMP4: Bone morphogenetic protein 4; BrdU: Bromodeoxyuridine; CD: Cluster of differentiation; cDNA: Complimentary deoxyribonucleic acid; CFU-F: Colony-forming unit fibroblast; COLL2: Collagen 2; EDTA: Ethylenediaminetetraacetic acid; ELISA: Enzyme-linked immuno sorbent assay; ESCs: Embryonic stem cells; GvHD: Graft-*versus*-host disease; HGF: Hepatocyte growth factor; HLA: Human leucocyte antigen; GAPDH: Glyceraldehyde 3-phosphate dehydrogenase; IDO: Indoleamine 2,3-dioxygenase; IFNγ-R1: Interferon γ-receptor 1; IL-: Interleukin-; LIF: Leukemia inhibitory growth factor; LPL: Lipoprotein lipase; MEM: Minimal essential medium; MSCA: Mesenchymal stem cell antigen; MSCs: Mesenchymal Stromal Cells; mRNA: Messenger ribonucleic acid; MS: Multiple sclerosis; NGFB: Nerve growth factor; Oct4: Octamer-binding transcription factor 4; OPN: Osteopontin; P: Passage; PBS: Phosphate buffered saline; PCR: Polymerase chain reaction; PDGFR: Platelet-derived growth factor receptor; PDT: Population doubling time; PD(L)-1: Programmed death (ligand)1; PE: Phycoerythrin; PHS: Pooled human serum; PPAR-γ: Peroxisome proliferator-activated receptor-γ; Prdm14: PR domain containing 14; SCM: Standard culture medium; SD: Standard deviation; Sox2/9: SRY (sex determining region Y)-box 2/9; SSEA-4: Stage-specific embryonic antigen 4; T1D: Type-1 diabetes; VEGF-A: Vascular endothelial growth factor-A.

## Competing interests

The authors declare no competing interests.

## Authors’ contributions

GS contributed to the conception and design of the study and to manuscript writing, and was responsible for collection of data. TK and KB were involved in the conception and design of the study, provision of the study material and the collection of data.. UHK collected the data. HN provided administrative support and helped with manuscript writing. RS was responsible for the conception and design of the study, assembled, analyzed and interpreted data, and wrote the manuscript. All authors read and approved the final manuscript.

## Pre-publication history

The pre-publication history for this paper can be accessed here:

http://www.biomedcentral.com/1741-7015/11/146/prepub

## Supplementary Material

Additional file 1: Figure S4 Flow cytometry gating strategy. FSC–SSC gating to separate debris from intact cells (G1). Dead cells were excluded by uptake of 7-AAD (G2 on 7-AAD negative (= live) cells). Percentage analysis of antigen-positive cells and fluorescence intensity was performed with FlowJo-7.2.5 software. For compensation of unspecific antibody binding, the positivity of the respective matched isotype control was subtracted from all samples. Click here for file

Additional file 2: Figure S5 Flow cytometry density plots. Density plots from flow cytometric analysis show a representative BM-MSC preparation (P1). Overlay density plots show gated cells after excluding non-viable cells and debris. Green: specific antibody; red: isotype control. Click here for file

Additional file 3: Table S1 Donor distribution and data for CFU-F analyses. Click here for file

Additional file 4: Figure S1 Expression of androgen receptor and FGFR3 by BM-MSCs. No difference was detected in androgen receptor and FGFR3 expression between BM-MSC preparations of female and male donors (**A, B**) and no significant correlation was found between these receptors and donor age (**C, D**) (n = 14 (7 female, 7 male)). Lower detection limits (ELISA): Androgen receptor: 113 pg/ml; FGFR3: 55 pg/ml. Two-tailed Student’s *t*-test and Spearman two-tailed correlation test, error bars: SD. Click here for file

Additional file 5: Figure S2 Adipogenic differentiation potential of MSCs. The percentage of CD10^+^ cells (n = 27) and the specific antibody mediated fluorescence per cell (ΔGeo Mean) of CD119 (n = 22) correlated positively with the induction of *PPARγ* mRNA under adipogenic differentiation (**A-D**). Moreover, the percentage of GD2^+^ cells and the specific antibody mediated fluorescence per cell (ΔGeo Mean) of GD2 correlated negatively with the induction of *LPL* mRNA under adipogenic differentiation (n = 26) (**E, F**). Hereby, we identified two phenotypes with either higher (CD10, CD119) or lower (GD2) adipogenic differentiation potential within the BM-MSC preparations. Spearman two-tailed correlation test (*P <0.05; **P <0.01). Click here for file

Additional file 6: Figure S3 Secretion profile of MSC trophic factors. BM-MSCs secreted the highest concentrations of VEGF-A and HGF, followed by LIF, Angiopoietin-1, bFGF and NGFB (n = 11) (**A**). Correlation analyses of the secreted factors to markers potentially defining MSC subpopulations revealed a significant negative correlation for HGF secretion to the expression of CD71, CD140b and Galectin 1 (n = 11 except for Galectin 1 (n = 9)) (**B**); no positive correlation of the tested markers to the secretion of trophic factors was identified. Neither donor age nor gender affected the secretion of trophic factors (**C, D**); no correlation of the marker expression to the Angiopoietin-1 “(non-)secretor” status of the MSCs was identified (n = 11) (**E, F**). Lower detection limits (Luminex^®^ and ELISA): NGF-b: 3.9 pg/ml; LIF: 2.5 pg/ml; FGF-b: 13.2 pg/ml; VEGF-A: 11.2 pg/ml; HGF: 2.2 pg/ml; Angiopoietin-1: 3.45 pg/ml; BMP4: 1.04 pg/ml. ANOVA analysis of variance followed by Tukey`s Multiple Comparison Test, Two-tailed Student’s *t*-test and Spearman two-tailed correlation test (*P <0.05; **P <0.01; ***P <0.001). Error bars: SD. Click here for file

Additional file 7: Table S2 Donor variations and data of Angiopoietin-1 secretion. Click here for file
