# Supplementary material for: Phenotype, donor age and gender affect function of human bone marrow-derived mesenchymal stromal cells
Source: BMC Med. 2013 Jun 11;11:146. doi: 10.1186/1741-7015-11-146 (PMC3694028; doi:10.1186/1741-7015-11-146)
Supplement: Additional file 1: Figure S4 — Flow cytometry gating strategy. FSC–SSC gating to separate debris from intact cells (G1). Dead cells were excluded by uptake of 7-AAD (G2 on 7-AAD negative (= live) cells). Percentage analysis of antigen-positive cells and fluorescence intensity was performed with FlowJo-7.2.5 software. For compensation of unspecific antibody binding, the positivity of the respective matched isotype control was subtracted from all samples. [file 1741-7015-11-146-S1.pdf]

**Supplemental Figure 4**

Flow cytometry gating strategy

## G1

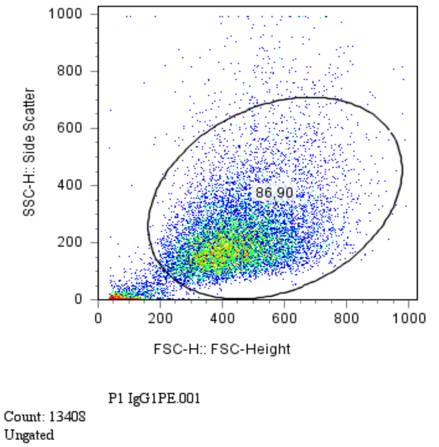


FSC-SSC gating to
separate debris from intact cells (G1).

## G2

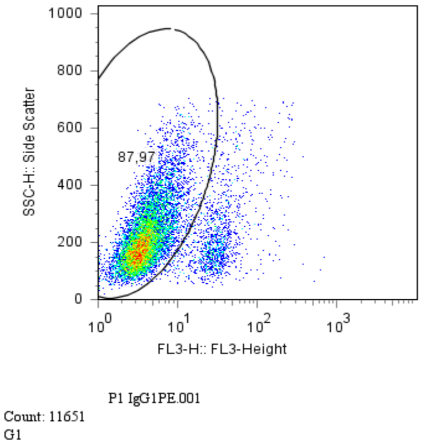


Exclusion of dead cells by uptake of 7-AAD
(G2 on 7-AAD negative [= live] cells).

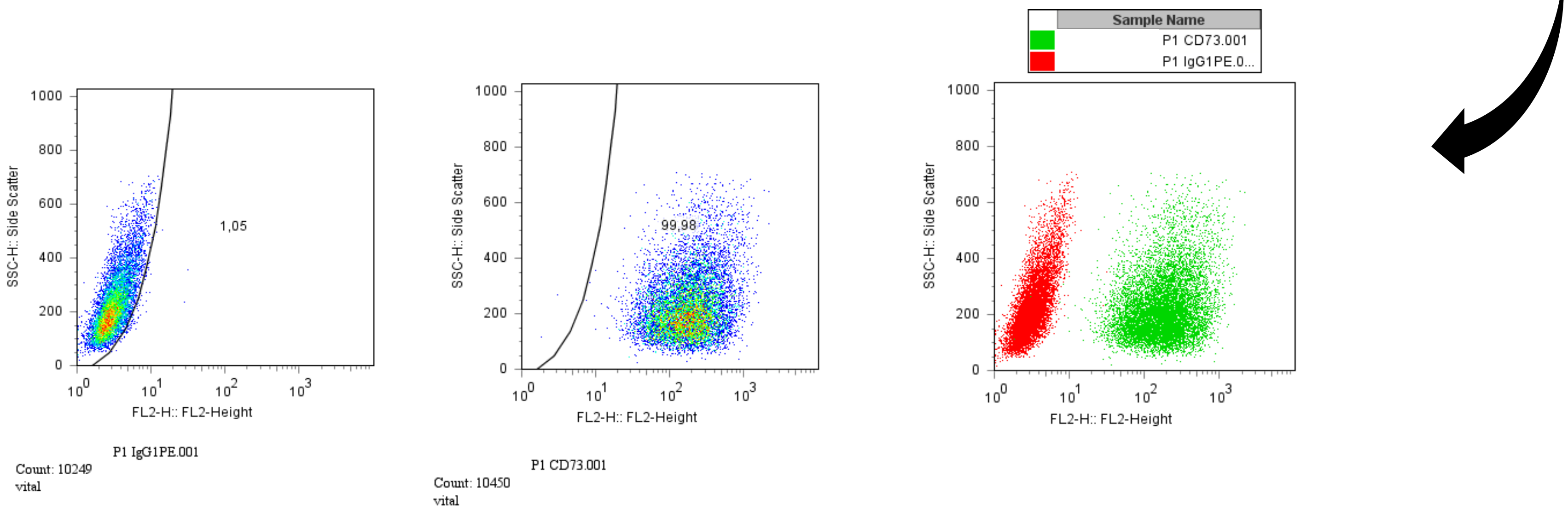


Analysis of percentage of antigen positive cells and fluorescence intensity. For compensation of unspecific antibody binding, the positivity of the respective matched isotype control was subtracted from all samples.
