# Supplementary material for: Phenotype, donor age and gender affect function of human bone marrow-derived mesenchymal stromal cells
Source: BMC Med. 2013 Jun 11;11:146. doi: 10.1186/1741-7015-11-146 (PMC3694028; doi:10.1186/1741-7015-11-146)

Supplemental Figure 5

Flow cytometry density plots

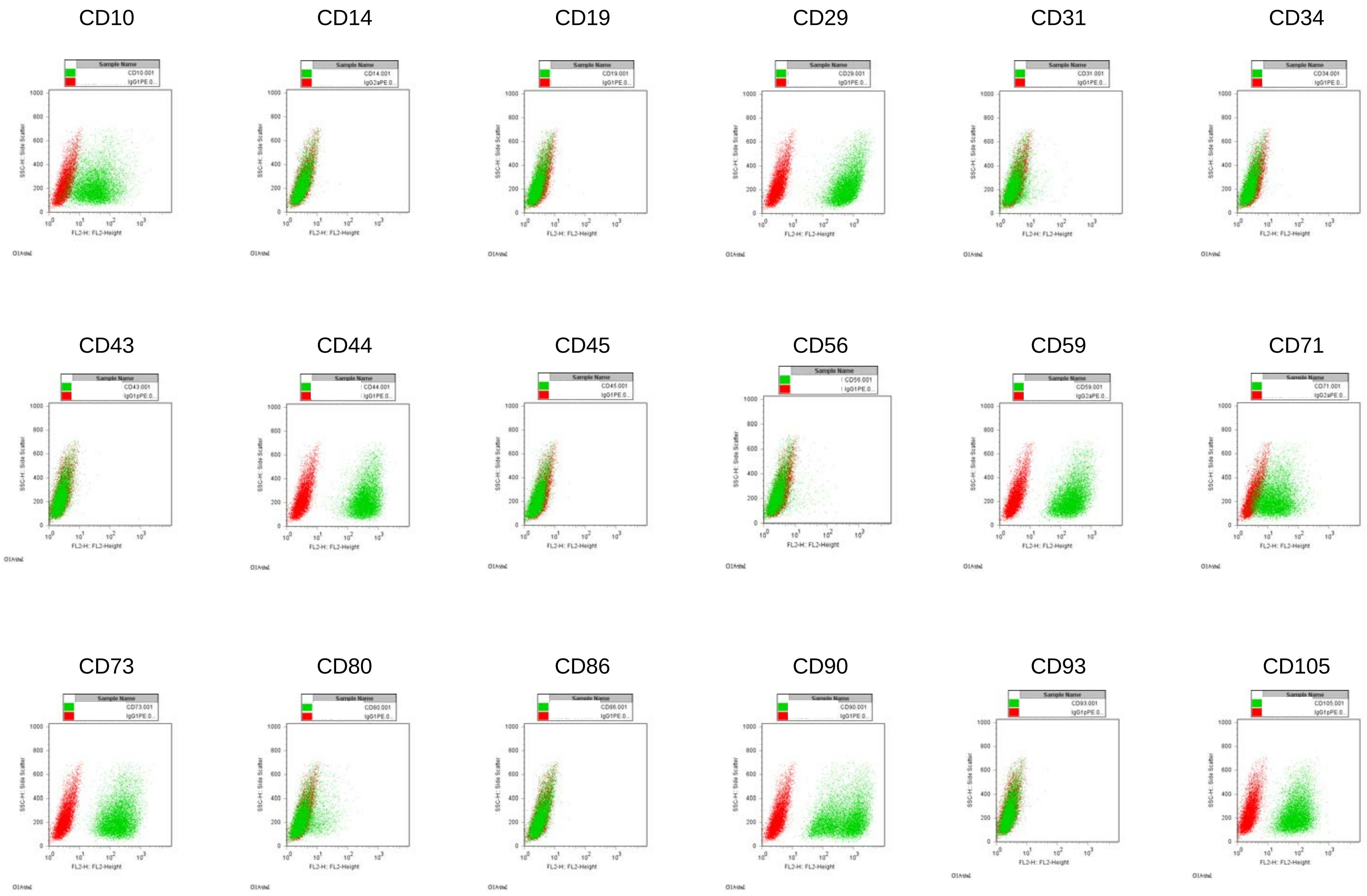

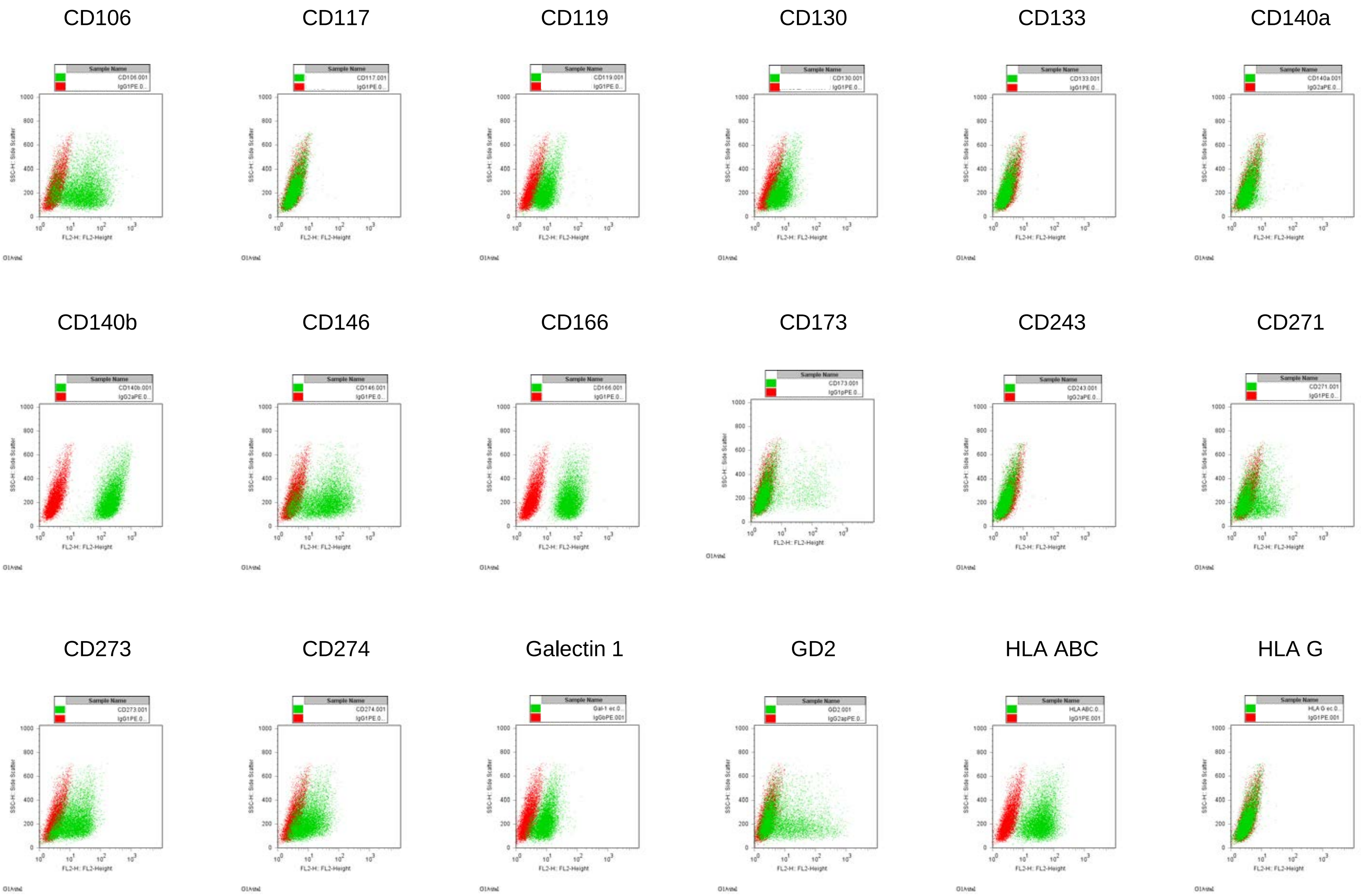
CD106
CD117
CD119
CD130
CD133
CD140a
CD140b
CD146
CD166
CD173
CD243
CD271
CD273
CD274
Galectin 1
GD2
HLA ABC
HLA G

SSEA-1

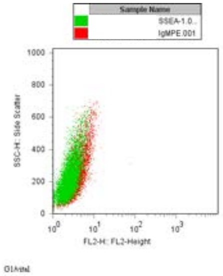

SSEA-4

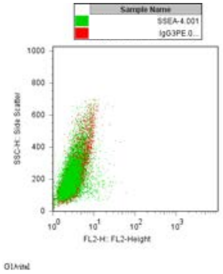

W8B2

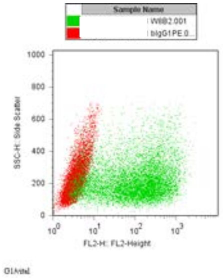

Supplement: Additional file 2: Figure S5 — Flow cytometry density plots. Density plots from flow cytometric analysis show a representative BM-MSC preparation (P1). Overlay density plots show gated cells after excluding non-viable cells and debris. Green: specific antibody; red: isotype control. [file 1741-7015-11-146-S2.pdf]
