# Supplementary material for: Phenotype, donor age and gender affect function of human bone marrow-derived mesenchymal stromal cells
Source: BMC Med. 2013 Jun 11;11:146. doi: 10.1186/1741-7015-11-146 (PMC3694028; doi:10.1186/1741-7015-11-146)
Supplement: Additional file 3: Table S1 — Donor distribution and data for CFU-F analyses. [file 1741-7015-11-146-S3.doc]

**Supplemental Table 1**

Donor distribution and data for CFU-F analyses

| **CFU-F (%)** | | | | | | | |
| --- | --- | --- | --- | --- | --- | --- | --- |
| **donor age** | **male** | **female** |  | **gender** | **donor age** | | |
| **<45 y** | 5.8 | 27.125 |  |  | **<45 y** | **45-65 y** | **>65 y** |
|  | 0.925 | 17 |  | **female** | 27.125 | 6.78333 | 6.55 |
|  | 16.15 | 16.125 |  |  | 17 | 2.125 | 11.51667 |
|  |  | 12 |  |  | 16.125 | 2.18333 | 6.2 |
|  |  | 6.95 |  |  | 12 | 7 | 3.133333 |
|  |  | 7.5 |  |  | 6.95 | 17.6667 | 5.566667 |
|  |  | 18.4 |  |  | 7.5 | 10.5 | 10.75 |
|  |  | 7.558333 |  |  | 18.4 | 4.46667 | 2.133333 |
| **45-65 y** | 4.366667 | 6.783333 |  |  | 7.5583 |  | 5.358333 |
|  | 1.383333 | 2.125 |  | **male** | 5.8 | 4.36667 | 1.983333 |
|  | 0.85 | 2.183333 |  |  | 0.925 | 1.38333 | 17.125 |
|  | 0 | 7 |  |  | 16.15 | 0.85 | 6.75 |
|  | 7.766667 | 17.66667 |  |  |  | 0 | 5.716667 |
|  | 0.8 | 10.5 |  |  |  | 7.76667 | 7.966667 |
|  | 3.7 | 4.466667 |  |  |  | 0.8 |  |
|  | 7.266667 |  |  |  |  | 3.7 |  |
|  | 0.966667 |  |  |  |  | 7.26667 |  |
|  | 8.133333 |  |  |  |  | 0.96667 |  |
|  | 1 |  |  |  |  | 8.13333 |  |
|  | 14 |  |  |  |  | 1 |  |
|  | 14.76667 |  |  |  |  | 14 |  |
|  | 3.916667 |  |  |  |  | 14.7667 |  |
|  | 7.1 |  |  |  |  | 3.91667 |  |
|  | 1.833333 |  |  |  |  | 7.1 |  |
|  | 4.783333 |  |  |  |  | 1.83333 |  |
|  | 8.383333 |  |  |  |  | 4.78333 |  |
|  | 5.008333 |  |  |  |  | 8.38333 |  |
| **>65 y** | 1.983333 | 6.55 |  |  |  | 5.00833 |  |
|  | 17.125 | 11.51667 |  |  |  |  |  |
|  | 6.75 | 6.2 |  |  |  |  |  |
|  | 5.716667 | 3.133333 |  |  |  |  |  |
|  | 7.966667 | 5.566667 |  |  |  |  |  |
|  |  | 10.75 |  |  |  |  |  |
|  |  | 2.133333 |  |  |  |  |  |
|  |  | 5.358333 |  |  |  |  |  |
