# Supplementary figures and images for: Phenotype, donor age and gender affect function of human bone marrow-derived mesenchymal stromal cells
Source: BMC Med. 2013 Jun 11;11:146. doi: 10.1186/1741-7015-11-146 (PMC3694028; doi:10.1186/1741-7015-11-146)

Supplemental Figure 1

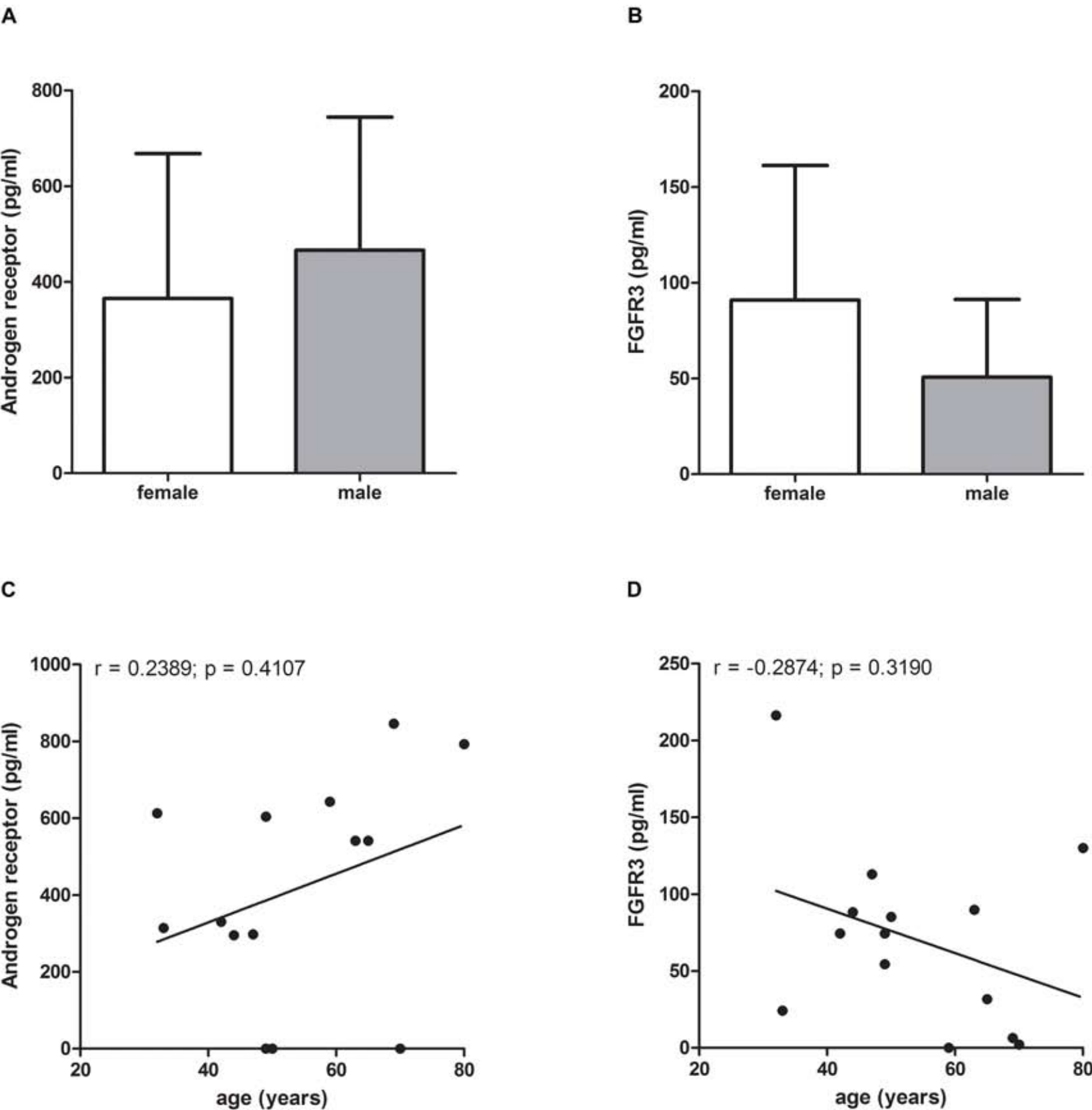

Supplement: Additional file 4: Figure S1 — Expression of androgen receptor and FGFR3 by BM-MSCs. No difference was detected in androgen receptor and FGFR3 expression between BM-MSC preparations of female and male donors (A, B) and no significant correlation was found between these receptors and donor age (C, D) (n = 14 (7 female, 7 male)). Lower detection limits (ELISA): Androgen receptor: 113 pg/ml; FGFR3: 55 pg/ml. Two-tailed Student’s t-test and Spearman two-tailed correlation test, error bars: SD. [file 1741-7015-11-146-S4.pdf]

Supplemental Figure 2

A

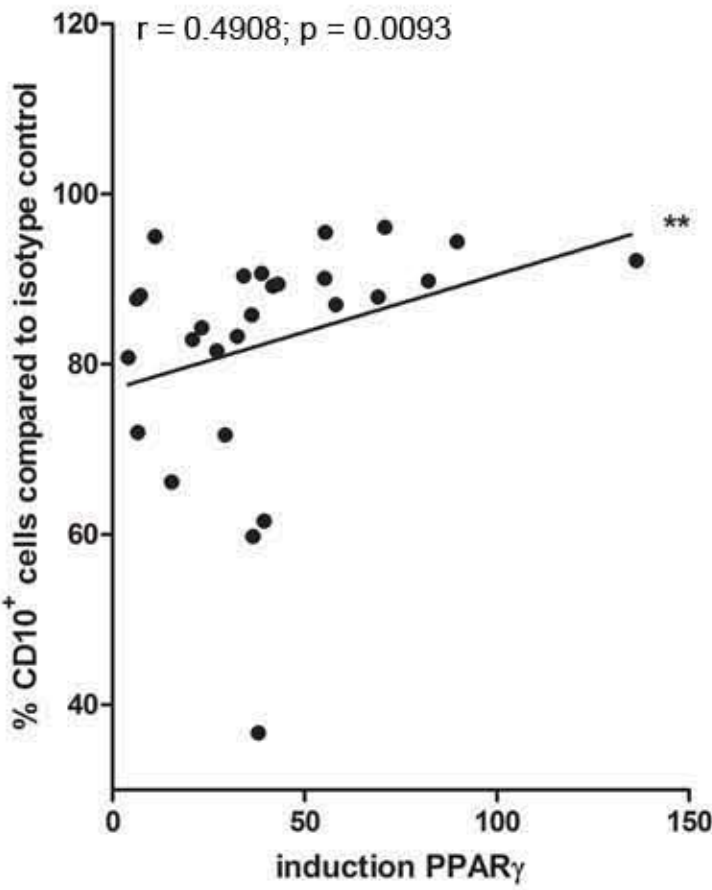

B

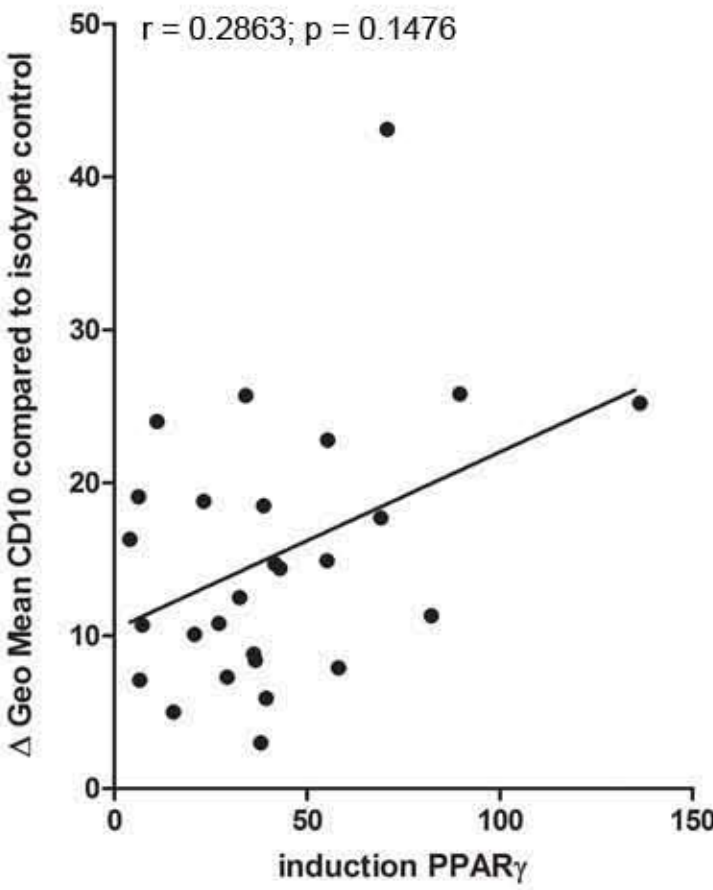

C

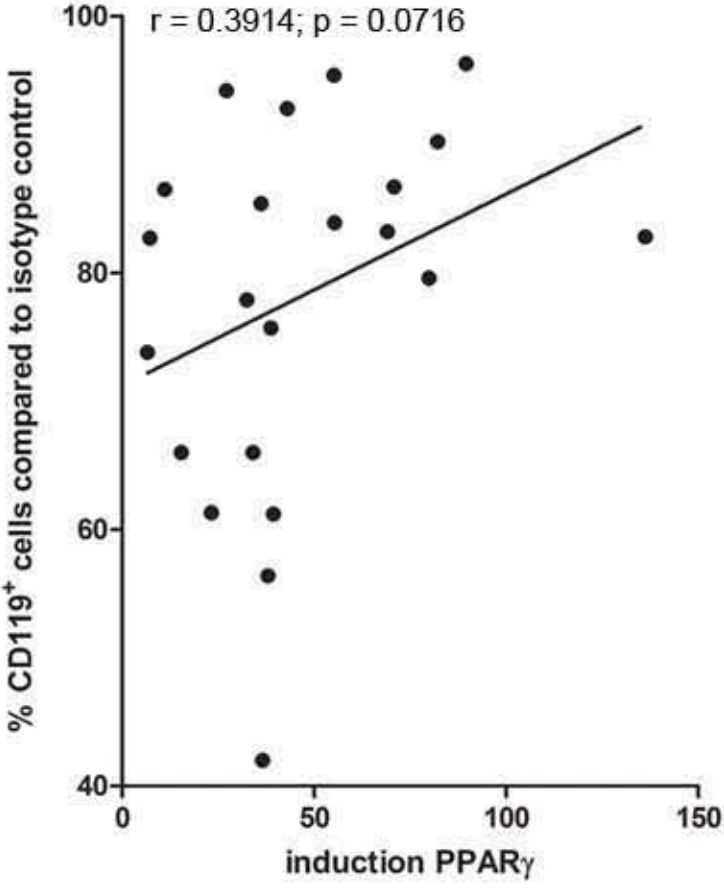

D

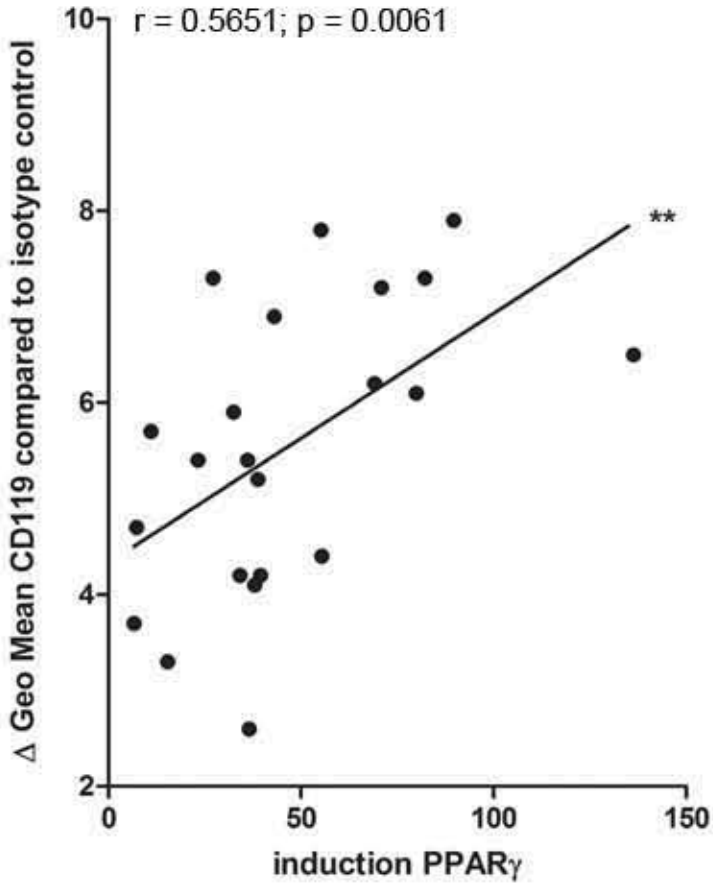

E

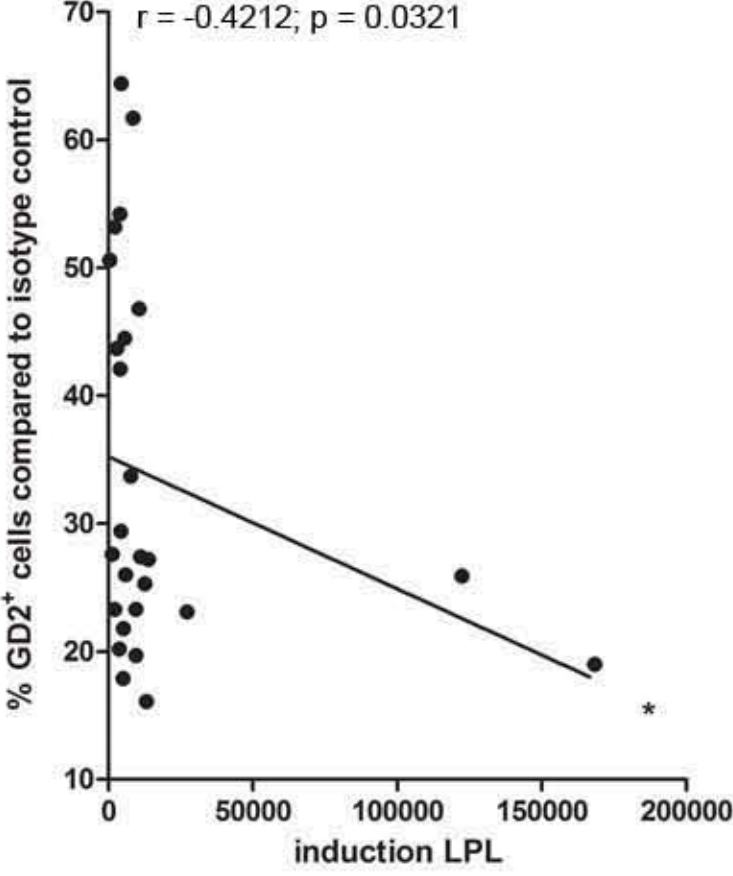

F

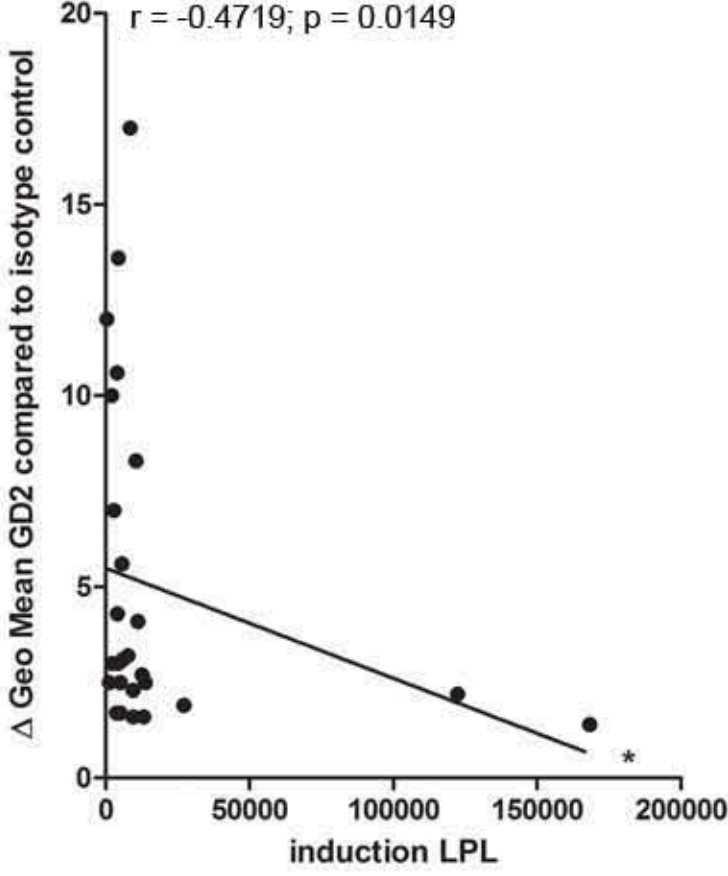

Supplement: Additional file 5: Figure S2 — Adipogenic differentiation potential of MSCs. The percentage of CD10+ cells (n = 27) and the specific antibody mediated fluorescence per cell (ΔGeo Mean) of CD119 (n = 22) correlated positively with the induction of PPARγ mRNA under adipogenic differentiation (A-D). Moreover, the percentage of GD2+ cells and the specific antibody mediated fluorescence per cell (ΔGeo Mean) of GD2 correlated negatively with the induction of LPL mRNA under adipogenic differentiation (n = 26) (E, F). Hereby, we identified two phenotypes with either higher (CD10, CD119) or lower (GD2) adipogenic differentiation potential within the BM-MSC preparations. Spearman two-tailed correlation test (*P <0.05; **P <0.01). [file 1741-7015-11-146-S5.pdf]

### Supplemental Figure 3

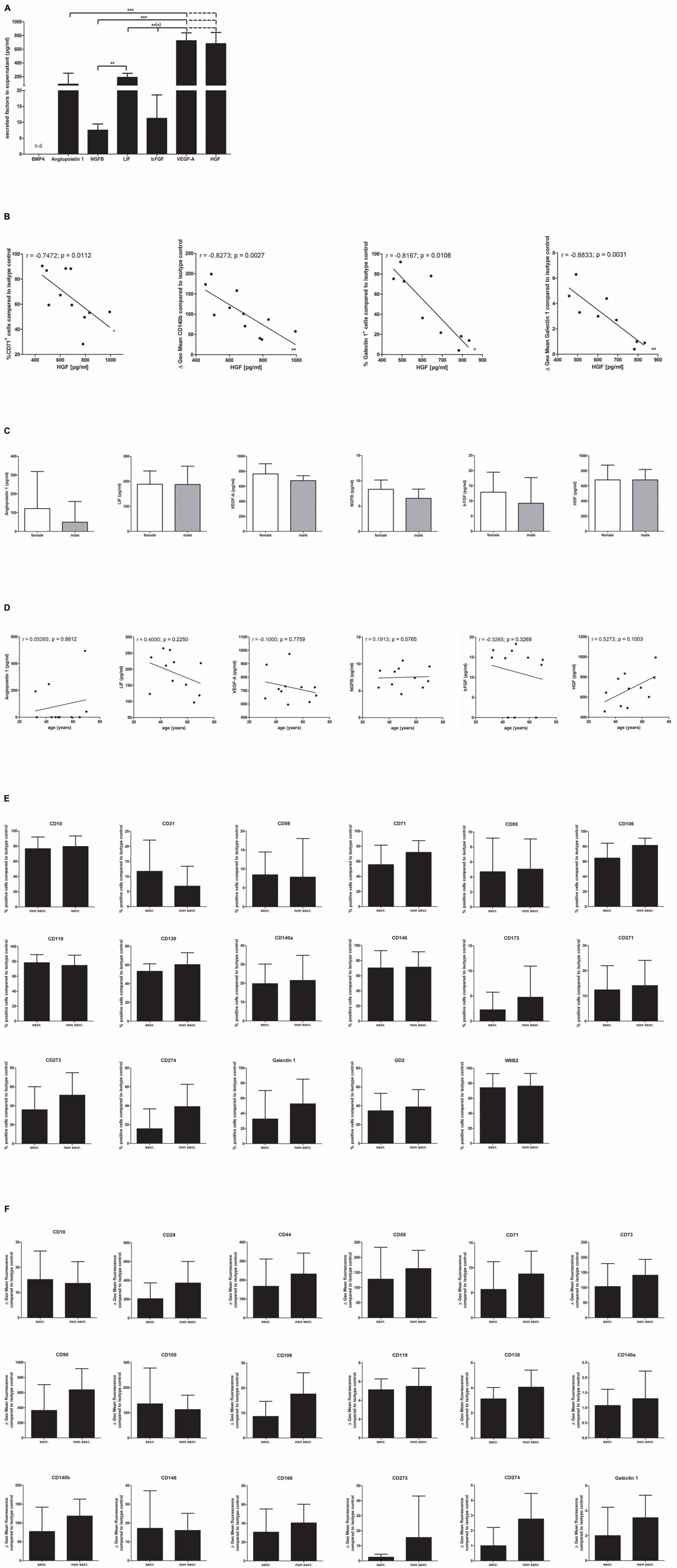

Supplement: Additional file 6: Figure S3 — Secretion profile of MSC trophic factors. BM-MSCs secreted the highest concentrations of VEGF-A and HGF, followed by LIF, Angiopoietin-1, bFGF and NGFB (n = 11) (A). Correlation analyses of the secreted factors to markers potentially defining MSC subpopulations revealed a significant negative correlation for HGF secretion to the expression of CD71, CD140b and Galectin 1 (n = 11 except for Galectin 1 (n = 9)) (B); no positive correlation of the tested markers to the secretion of trophic factors was identified. Neither donor age nor gender affected the secretion of trophic factors (C, D); no correlation of the marker expression to the Angiopoietin-1 “(non-)secretor” status of the MSCs was identified (n = 11) (E, F). Lower detection limits (Luminex® and ELISA): NGF-b: 3.9 pg/ml; LIF: 2.5 pg/ml; FGF-b: 13.2 pg/ml; VEGF-A: 11.2 pg/ml; HGF: 2.2 pg/ml; Angiopoietin-1: 3.45 pg/ml; BMP4: 1.04 pg/ml. ANOVA analysis of variance followed by Tukey`s Multiple Comparison Test, Two-tailed Student’s t-test and Spearman two-tailed correlation test (*P <0.05; **P <0.01; ***P <0.001). Error bars: SD. [file 1741-7015-11-146-S6.pdf]
