# Supplementary material for: Phenotype, donor age and gender affect function of human bone marrow-derived mesenchymal stromal cells
Source: BMC Med. 2013 Jun 11;11:146. doi: 10.1186/1741-7015-11-146 (PMC3694028; doi:10.1186/1741-7015-11-146)
Supplement: Additional file 7: Table S2 — Donor variations and data of Angiopoietin-1 secretion. [file 1741-7015-11-146-S7.doc]

**Supplemental Table 2**

Donor variations and data of Angiopoietin-1 secretion.

| **sample number** | **age** (y) | **gender** | **Angiopoietin-1 in supernatant** [pg/ml] |
| --- | --- | --- | --- |
| 20 | 50 | f | n.d. |
| 21 | 70 | f | 41.87 |
| 22 | 44 | f | n.d. |
| 23 | 59 | m | n.d. |
| 24 | 32 | f | 193.05 |
| 25 | 47 | m | n.d. |
| 26 | 49 | m | n.d. |
| 27 | 65 | m | n.d. |
| 28 | 69 | f | 494.79 |
| 29 | 33 | f | n.d. |
| 30 | 42 | m | 246.60 |

n.d. = not detectable; detection limit: 3.45 pg/ml
